# Physiotherapy Regimens in Esophagectomy and Gastrectomy: a Systematic Review and Meta-Analysis

**DOI:** 10.1245/s10434-021-11122-7

**Published:** 2021-12-27

**Authors:** Karina H. Tukanova, Swathikan Chidambaram, Nadia Guidozzi, George B. Hanna, Alison H. McGregor, Sheraz R. Markar

**Affiliations:** 1 Department of Surgery and Cancer, Imperial College London, London, UK; 2 Department of Surgery, University of the Witwatersrand, Johannesburg, South Africa; 3 Nuffield Department of Surgery, University of Oxford, Oxford, UK; 4 Department of Molecular Medicine and Surgery, Karolinska Institutet, Solna, Sweden; 5 Division of Surgery, Department of Surgery and Cancer, St Mary’s Hospital, London, UK

## Abstract

**Background:**

Esophageal and gastric cancer surgery are associated with considerable morbidity, specifically postoperative pulmonary complications (PPCs), potentially accentuated by underlying challenges with malnutrition and cachexia affecting respiratory muscle mass. Physiotherapy regimens aim to increase the respiratory muscle strength and may prevent postoperative morbidity.

**Objective:**

The aim of this study was to assess the impact of physiotherapy regimens in patients treated with esophagectomy or gastrectomy.

**Methods:**

An electronic database search was performed in the MEDLINE, EMBASE, CENTRAL, CINAHL and Pedro databases. A meta-analysis was performed to assess the impact of physiotherapy on the functional capacity, incidence of PPCs and postoperative morbidity, in-hospital mortality rate, length of hospital stay (LOS) and health-related quality of life (HRQoL).

**Results:**

Seven randomized controlled trials (RCTs) and seven cohort studies assessing prehabilitation totaling 960 patients, and five RCTs and five cohort studies assessing peri- or postoperative physiotherapy with 703 total patients, were included. Prehabilitation resulted in a lower incidence of postoperative pneumonia and morbidity (Clavien–Dindo score ≥ II). No difference was observed in functional exercise capacity and in-hospital mortality following prehabilitation. Meanwhile, peri- or postoperative rehabilitation resulted in a lower incidence of pneumonia, shorter LOS, and better HRQoL scores for dyspnea and physical functioning, while no differences were found for the QoL summary score, global health status, fatigue, and pain scores.

**Conclusion:**

This meta-analysis suggests that implementing an exercise intervention may be beneficial in both the preoperative and peri- or postoperative periods. Further investigation is needed to understand the mechanism through which exercise interventions improve clinical outcomes and which patient subgroup will gain the maximal benefit.

**Supplementary Information:**

The online version contains supplementary material available at 10.1245/s10434-021-11122-7.

## Background

Despite improvements in perioperative management, surgery for esophageal and gastric cancer is associated with considerable morbidity, particularly postoperative pulmonary complications (PPCs). PPCs include pneumonia and atelectasis, occur in about 20–40% of patients,^1–3^ and account for up to 55% of in-hospital deaths.^4,5^ The risk for PPCs development is multifactorial and includes both patient- and treatment-related factors. Open surgery is associated with significantly higher pain scores, which may interfere with respiratory mechanics and impede adequate mobilization, resulting in atelectasis and shallow breathing.^6–10^ Other intraoperative procedures that might contribute to PPCs include mechanical ventilation, patient positioning, and administration of sedatives.^11–14^ Following surgery, patients undergoing esophagogastrectomy often require chest drainage, contributing further to postoperative pain and altered respiratory mechanics.^15,16^

Preoperative factors also have an impact on the development of PPCs, including preoperative chemoradiotherapy. Although neoadjuvant chemoradiotherapy has led to a significant improvement in survival in esophageal and esophagogastric junction cancer,^17^ several studies suggest that the associated toxicity affects pulmonary function, causing a decline in diffusion capacity, and total lung and vital capacity.^18^ In addition to decreased exercise capacity following surgery,^19^ neoadjuvant chemotherapy is also associated with reduced physical fitness.^20^ Other predictive factors for severe complications include lower preoperative forced expiratory volume in 1 second (FEV_1_),^21,22^ decreased diffusion capacity,^23^ multiple comorbidities, and smoking.^24^ Physical activity levels also play an important role in the postoperative period, as patients with higher activity levels appeared to have a significantly lower risk for cancer recurrence, and had higher overall survival rates and better HRQoL scores compared with inactive participants.^25^ Finally, patients with gastrointestinal cancers often present with malnutrition and cachexia^26–28^ affecting respiratory muscle mass and strength, and subsequently increasing the risk for development of PPCs^29,30^ and poor functional capacity.^31^

Physiotherapy regimens such as early mobilization and breathing exercises aim to decrease the risk for PPCs by reversing atelectasis.^32^ There is some evidence that breathing exercises, both in the preoperative period^32^ and during postoperative recovery,^33^ decrease the incidence of PPCs in upper abdominal surgery. However, due to insufficient strong evidence, routine implementation of respiratory physiotherapy following abdominal surgery has not yet been implemented as a standard of care.^32^

To date, there has been no published meta-analysis assessing the effect of prehabilitation and peri- or postoperative physiotherapy regimens on postoperative mortality and morbidity in esophageal and gastric cancer surgery. The primary aim of this meta-analysis was to assess the impact of physiotherapy regimens on the incidence of major postoperative morbidity and in-hospital mortality. The secondary aims were to assess whether physiotherapy implementation decreases the length of hospital stay (LOS) and improves the functional exercise capacity and HRQoL.

## Methods

### Search Strategy

A literature search was performed on the 18 February 2021 to identify relevant studies assessing physiotherapy regimens in patients undergoing esophagectomy or gastrectomy in the MEDLINE (Ovid), EMBASE (Ovid), Cochrane Central Register of Controlled Trials, CINAHL, and Physiotherapy Evidence (Pedro) databases. The search included the following index or free-text words, including synonyms and closely related words: ‘(o)esophagectomy’, ‘gastrectomy’, ‘physiotherapy’, ‘physical therapy’, ‘kinesi(o)therapy’, ‘muscle training’, ‘mobilization’, and ‘breathing techniques’. References of included articles were screened and a hand-search was performed to identify missing articles. The full electronic search strategy is available in electronic supplementary (ES) Table S1.

Two reviewers (KHT and NG) independently assessed the titles and abstracts for inclusion of relevant references. In the case of disagreement for inclusion, a third author (SRM) was consulted. Authors of the included studies were contacted to locate unpublished data.

The Preferred Reporting Items for Systematic Reviews and Meta-Analyses (PRISMA)^33^ guidelines were followed (ES Table S2).

### Study Selection

Randomized controlled trials (RCTs), quasi-randomized trials, and cohort studies were included, implementing physiotherapy regimens in the pre-, peri- or postoperative periods for patients who have undergone esophagectomy or gastrectomy for malignant disease, either by open surgery or a minimally invasive approach.

Comparative studies were excluded if no outcome data were provided for the control or intervention groups. Studies were excluded if physiotherapy in the form of early mobilization was part of an enhanced recovery pathway and the impact of the pathway was assessed without evaluating the impact of the physiotherapy component.

### Outcome Measures

The primary outcomes were the incidence of postoperative morbidity assessed using a Clavien–Dindo classification^34^ (CDC) of 2 or higher, PPCs, and in-hospital mortality, while the secondary outcomes were assessment of the functional capacity via the 6-minute walking test (6MWT), LOS, and HRQoL.

### Quality Assessment of Selected Studies

Two reviewers (KT and NG) assessed the quality of each included study by independently evaluating the risk of bias using the revised Cochrane risk-of-bias tool (RoB2)^35^ for the assessment of randomized trials and the Newcastle–Ottawa Scale (NOS)^36^ for the assessment of non-randomized studies. The RoB2 tool categorizes the risk of bias into ‘low’, ‘some concerns’, or ‘high’ risk of bias. For the NOS scores ranging from 0 to 9, we considered a score of 0–3, 4–6, and 7–9 as low, moderate, or high-quality studies, respectively.

### Statistical Analysis

Meta-analysis of the data was performed using the Review Manager version 5.3 software (Cochrane Collaboration, Oxford, UK). Both the fixed-effects and random-effects models were considered in the analysis of the data and were the most appropriate models used to pool the results based on the distribution of the data. The standard heterogeneity test, the *I*^2^ statistic, was used to assess the consistency of the effect sizes, which indicates the percentage of the variability in effect estimates because of true between-study variance rather than within-study variance. Statistical heterogeneity was graded as low, moderate, or high, with an *I*^2^ of above 25%, 50%, and 75%, respectively.^37^

## Results

### Literature Search Results

**Fig. 1:**
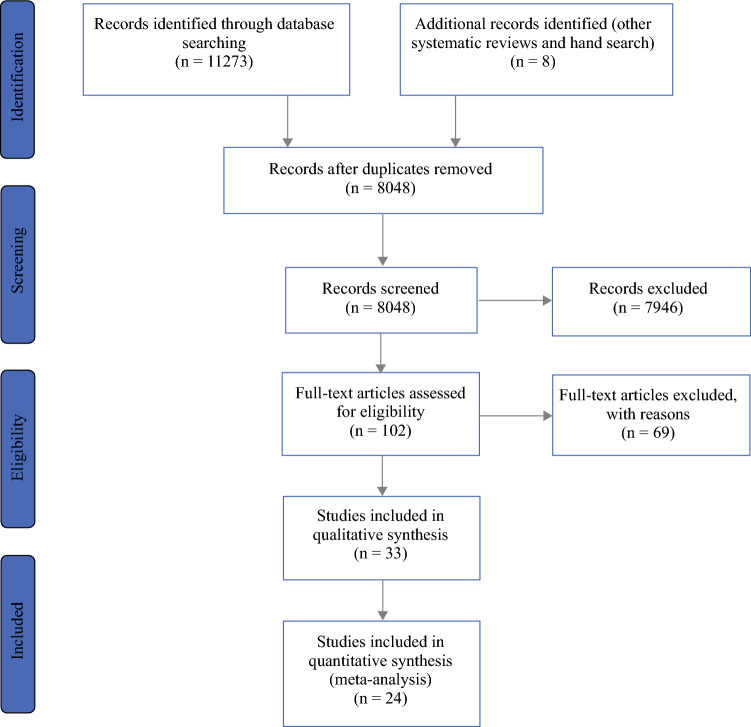
PRISMA flow chart. *PRISMA* Preferred Reporting Items for Systematic Reviews and Meta-Analyses

The electronic database searches yielded 11,273 results. The database searches were complemented by a hand search, identifying eight articles through published systematic reviews and protocols. After removal of duplicates, 8048 publications were screened on the abstract. Subsequent screening of the full-text identified 33 relevant records, 17 of which assessed preoperative exercise programs (prehabilitation), while 16 studies assessed peri- or postoperative physiotherapy regimens (Fig. 1).

Among the studies assessing prehabilitation, seven were RCTs^38–44^ with an intervention group and a comparative control group, while one RCT^45^ compared two different types of interventions; the remaining nine publications were cohort studies.^2,46–53^ For the studies assessing peri- and postoperative physiotherapy, there were six RCTs^54–59^ comparing an intervention with a control group, and one study^60^ compared two different types of physiotherapy. Among the cohort studies, eight studies^61–68^ had an intervention group and a comparative control, and one study^69^ assessed three groups consisting of two physiotherapy intervention groups and one comparative control group. An overview of interventions for studies included in the meta-analysis is shown in Table 1; a summary of the study characteristics of all studies is shown in ES Table S3, with a comprehensive summary of interventions for all studies provided in shown in ES Table S4. A summary of the statistical method used for each analysis is presented in ES Table S5.

**Table 1:** Overview of physiotherapy interventions of the studies included in the meta-analysis

Authors (year)	Study type	Group (*n*)	Intervention timing and duration	Type of intervention	Outcomes
*Prehabilitation*					
Dettling et al.^52^ (2013)	NRS	IG (44)	Preoperative respiratory training (duration: minimum 2 weeks) and postoperative physical therapy (duration: 10 days)	Preoperative IMT Postoperative respiratory rehabilitation and mobilization	Incidence pneumonia: 11/44 Incidence other PPCs: 9/44 Incidence in-hospital mortality: 1/44 Median (IQR) LOS: 13.5 (10.0–22.75)
		CG (39)	–	Postoperative respiratory rehabilitation and mobilization	Incidence pneumonia: 9/39 Incidence other PPCs: 6/39 Incidence in-hospital mortality: 3/39 Median (IQR) LOS: 12.0 (9.0–14.0)
Inoue et al.^2^ (2013)	Cohort study	IG (63)	Preoperatively (duration: minimum 1 week)	Preoperative IMT, muscle strength training for UL and LL, and abdominal muscles and aerobic exercise Postoperative respiratory rehabilitation and mobilization	Incidence pneumonia: 4/63 Incidence CDC ≥ II: 4/63 Mean (SD) LOS: 41.2 (32.2)
		CG (37)	–	Preoperative rehabilitation insufficiently (or not) received Postoperative respiratory rehabilitation and mobilization	Incidence pneumonia: 9/37 Incidence CDC ≥ II: 9/37 Mean (SD) LOS: 49.8 (28.9)
Cho et al.^49^ (2014)	Cohort study	IG (18)	Preoperatively (duration: 4 weeks)	Aerobic exercise and stretching before and after exercise Resistance training	Incidence pneumonia: 0/18 Incidence other PPCs: 3/18 Median (95% CI) LOS: 9.0 (9.0–10.0)
		CG (54)	–	No preoperative intervention	Incidence pneumonia: 2/54 Incidence other PPCs: 8/54 Median (95% CI) LOS: 10.0 (9.0–11.0)
Yamana et al.^38^ (2015)	RCT	IG (30)	Preoperatively (duration: minimum 1 week)	Preoperative IMT, muscle strength exercises LL and abdominal muscles, and aerobic exercise Postoperative respiratory rehabilitation and mobilization	Incidence pneumonia: 10/30 Incidence CDC ≥ II: 8/30 Incidence in-hospital mortality: 0/30
		CG (30)	–	No preoperative intervention Postoperative respiratory rehabilitation and mobilization	Incidence pneumonia: 17/30 Incidence CDC ≥ II: 18/30 Incidence in-hospital mortality: 0/30
Weblin et al.^48^ (2017)	NRS	IG (13)	Preoperatively (duration: 4 weeks)	Preoperative respiratory rehabilitation with warm-up, cool down and mobilization ERAS and enhanced postoperative respiratory rehabilitation and mobilization	Median (IQR) LOS: 13.0 (11.0–20.0)
		CG (10)	–	ERAS and enhanced postoperative respiratory rehabilitation and mobilization	Median (IQR) LOS: 14.0 (11.5–21.0)
Valkenet et al.^44^ (2017)	RCT	IG (120)	Preoperatively (duration: minimum 2 weeks)	Preoperative IMT Postoperative airway clearance technique and early mobilization	Incidence pneumonia: 47/120 Incidence other PPCs: 41/118 Incidence in-hospital mortality: 5/120 Mean (SD) LOS: 18.4 (8.0)
		CG (121)	–	No preoperative intervention Postoperative airway clearance technique and early mobilization	Incidence pneumonia: 43/121 Incidence other PPCs: 40/120 Incidence in-hospital mortality: 3/121 Mean (SD) LOS: 20.5 (20.9)
Christensen et al.^46^ (2019)	NRS	IG (21)	Preoperatively during neoadjuvant treatment (duration: 9 weeks)	Aerobic and resistance training	Incidence pneumonia: 4/21 Incidence CDC ≥ II: 11/21 Median (IQR) LOS: 10.0 (9.0–11.0)
		CG (29 )	–	Information advice, physiotherapy guidelines	Incidence pneumonia: 3/29 Incidence CDC ≥ II: 13.29 Median (IQR) LOS: 9.0 (8.0–11.0)
Minnella et al.^40^ (2018)	RCT	IG (26)	Preoperatively, duration NS (77% during NACT)	Aerobic and muscle strength exercises Nutritional advice and support	Mean (SD) postop 6MWT: 467.5 (65.6) Incidence CDC ≥ II: 12/24 Incidence in-hospital mortality: 0/26 Median (IQR) LOS: 8.0 (5.75–11.75)
		CG (25)	–	Standardized ERAS with respiratory physiotherapy and early mobilization	Mean (SD) postop 6MWT: 367.4 (87.0) Incidence CDC ≥ II: 18/25 Incidence in-hospital mortality: 2/25 Median (IQR) LOS: 7.0 (5.5–12.5)
Guinan et al.^42^ (2019)	RCT	IG (28)	Subcohort PREPARE trial, intervention as described by Valkenet et al.^44^		Mean (SD) postop 6MWT: 305.6 (116.3) Incidence PPCs: 9/28 Incidence in-hospital mortality: 0/28 Median (IQR) LOS: 17.0 (8.0)
		CG (32)	–	No preoperative intervention Postoperative airway clearance technique and early mobilization	Mean (SD) postop 6MWT: 380.2 (47.1) Incidence PPCs: 11/32 Incidence in-hospital mortality: 0/32 Median (IQR) LOS: 18.0 (15.0)
Lam et al.^41^ (2018)	RCT, thesis	IG (5)	Preoperatively (duration: 14–16 weeks)	In-hospital: aerobic and muscle strengthening exercise Home-based IMT	Incidence PPCs: 4/5
		CG (6)	–	Home exercise advice	Incidence PPCs: 4/6
Akiyama et al.^51^ (2021)	Cohort study	IG (23)	Preoperatively (duration: 1 week)	In-hospital: aerobic exercise and muscle strength training Home-based: 1-month preoperative IMT, mobilization and muscle strength training Postoperative early mobilization, ambulation, IMT and training for chewing and swallowing and aerobic exercise	Mean (SD) postop 6MWT: 431.5 (80.0) Incidence pneumonia: 1/23 Incidence other PPCs: 1/23 Incidence CDC ≥ II: 2/23 Incidence in-hospital mortality: 0/23 Median (IQR) LOS: 14.0 (12.0–16.0)
		CG (25)	–	Historical controls: Home-based: 1-month preoperative IMT, mobilization and muscle strength training Postoperative early mobilization, ambulation, IMT and training for chewing and swallowing and aerobic exercise	Mean (SD) postop 6MWT: 378.0 (68.7) Incidence pneumonia: 5/25 Incidence other PPCs: 9/25 Incidence CDC ≥ II: 8/25 Incidence in-hospital mortality: 0/25 Median (IQR) LOS: 16.0 (13.5–19.5)
Halliday et al.^47^ (2020)	Cohort study	IG (38)	Preoperatively during neoadjuvant treatment, duration NS	Aerobic and strength exercise training Nutritional support Psychological support ERAS with early mobilization	Incidence pneumonia: 10/38 Incidence other PPCs: 12/38 Incidence CDC ≥ II: 12/38 Median (IQR) LOS: 10.0 (8.0–17.0)
		CG (38)	–	ERAS with early mobilization	Incidence pneumonia: 25/38 Incidence other PPCs: 26/38 Incidence CDC ≥ II: 18.38 Median (IQR) LOS: 13.0 (11.0–20.0)
Swaminathan et al.^43^ (2020)	RCT	IG (29)	Preoperatively (duration: 1 week)	IMT with IS ERAS protocol with early mobilization	Incidence CDC ≥ II: 1/29 Median (IQR) LOS: 11.0 (3.0)
		CG (29)	–	No preoperative intervention	Incidence CDC ≥ II: 4/29 Median (IQR) LOS: 13.0 (4.0)
Zylstra et al.^53^ (2020)	Cohort	IG (13)	Preoperatively during neoadjuvant treatment, duration NS	Aerobic and strength training Core strength and stability training Flexibility exercises	Incidence PPCs: 4/13 Incidence CDC ≥ II: 2/13 Incidence in-hospital mortality: 0/13 Median (IQR) LOS: 10.83 (9.0–13.0)
		CG (14)	–	No preoperative intervention	Incidence PPCs: 4/14 Incidence CDC ≥ II: 3/14 Incidence in-hospital mortality: 0/14 Median (IQR) LOS: 9.67 (8.0–12.0)
*Peri- or postoperative rehabilitation*					
Lunardi et al.^67^ (2011)	Cohort study	IG (40)	Immediate postoperatively, duration NS (until discharge)	IMT, airway clearance maneuvers and early mobilization	Incidence pneumonia: 1/40 Incidence other PPCs: 1/40 Median (95% CI) LOS: 13.5 (3.6–29.8)
		CG (30)	–	Historical controls	Incidence pneumonia: 3/30 Incidence other PPCs: 1/30 Median (95% CI) LOS: 14.0 (8.0–24.2)
Lococo et al.^68^ (2012)	Cohort study	IG (8)	Postoperatively (duration: 4 weeks)	Aerobic exercise, IMT and muscle strength training for UL and LL Educational sessions for nutrition, psychological support and breathing exercises	Incidence PPCs: 2/8
		CG (50)	–	Historical controls: IMT, to achieve early mobilization General exercise therapy	Incidence PPCs: 13/50
Akiyama et al.^61^ (2017)	Cohort study	IG (31)	Perioperatively, duration NS	IMT, muscle strength training and early mobilization	Incidence pneumonia: 4/31 Incidence other PPCs: 14/31 Incidence in-hospital mortality: 0/31
		CG (21)	–	Historical controls	Incidence pneumonia: 5/21 Incidence other PPCs: 8/21 Incidence in-hospital mortality: 0/21
Fagevik Olsén et al.^55^ (2017)	RCT	IG (20)	Postoperatively, at discharge (duration: 3 months)	IMT with CPAP during ICU; deep breathing exercises with PEP; mobilization; muscle strength training Respiratory rehabilitation: stretching, IMT	Mean (SD) LOS: 19.7 (10.2) HRQoL: Mean Summary score (SD): 78.37 (7.43) Mean Global Health (SD): 61.6 (20.3) Mean Physical Functioning (SD): 85.3 (15.8) Mean Fatigue score (SD): 31.5 (20.3) Mean Pain score (SD): 18.5 (24.8) Mean Dyspnea score (SD): 33.3 (20.4)
		CG (23)	–	Advice given to avoid specific interventions during the first 3 postoperative months	Mean (SD) LOS: 18.3 (6.3) HRQoL: Mean Summary score (SD): 77.52 (11.5) Mean Global Health (SD): 70.1 (22.5) Mean Physical Functioning (SD): 78.8 (16.3) Mean Fatigue score (SD): 38.4 (28.2) Mean Pain score (SD): 20.5 (22.4) Mean Dyspnea score (SD): 42.4 (29.4)
Chen et al.^56^ (2017)	RCT	IG (39)	Immediate postoperatively, duration NS (until discharge)	Early mobilization Nutritional assistance: education, encourage oral intake and feeding assistance if needed	Median (IQR) LOS: 12.0 (6.0)
		CG (41)	–	Mobilization encouraged, not enforced	Median (IQR) LOS: 14.0 (9.0)
O'Neill et al. (2018)^58^	RCT	IG (21)	Postoperatively, in long-term survivors (duration: 12 weeks)	Aerobic exercise Resistance training	HRQoL: Mean Summary score (SD): 92.64 (11.20) Mean Global Health (SD): 79.17 (29.16) Mean Physical Functioning (SD): 93.33 (20.00) Mean Fatigue score (SD): 22.33 (11.00) Mean Pain score (SD): 0.00 (29.17) Mean Dyspnea score (SD): 0.00 (33.33)
		CG (22)	–	No postoperative intervention	HRQoL: Mean Summary score (SD): 96.36 (7.49) Mean Global Health (SD): 75.00 (16.66) Mean Physical Functioning (SD): 83.33 (26.67) Mean Fatigue score (SD): 22.33 (44.67) Mean Pain score (SD): 0.00 (33.33) Mean Dyspnea score (SD): 0.00 (33.33)
Jianjun et al.^62^ (2019)	Cohort study	IG (60)	Perioperatively (duration: 1 week)	Encouraged for preoperative IMT and endurance training Nutritional support if needed	Incidence pneumonia: 1/60 Mean (SD) LOS: 16.8 (3.5)
		CG (60)	–	Encouraged ambulation and dietary advice	Incidence pneumonia: 3/60 Mean (SD) LOS: 18.6 (4.1)
Wang et al.^65^ (2020)	Cohort study	IG (156) PSM (14)	Perioperatively, duration NS (until discharge)	Preoperative IMT, expiratory flow rate training; board training Postoperative early mobilization, IMT, airway clearance techniques and hand-assisted sputum excretion Administration of Ambroxol and Doxofylline	Incidence pneumonia: 7/14 Incidence other PPCs: 7/14
		CG (387) PSM (27)	–	No perioperative intervention	Incidence pneumonia: 20/27 Incidence other PPCs: 7/27
Jiao et al.^54^ (2020)	RCT	IG (43)	Perioperatively, duration NS	Preoperative IMT Postoperative sputum elimination, atomization inhalation if necessary Nutritional support: NG feeding during the first 3 days, gradually receiving high protein, high vitamin, high calorie and digestible food	Incidence pneumonia: 1/43 Incidence other PPCs: 1/43
		CG (43)	–	No perioperative intervention	Incidence pneumonia: 3/43 Incidence other PPCs: 4/43
van Vulpen et al.^59^ (2021)	RCT	IG (54)	Postoperatively (duration: 12 weeks)	Aerobic exercise Resistance training Warm-up and cool-down	HRQoL: Mean Summary score (SD): 86.52 (9.65) Mean Global Health (SD): 76.80 (15.90) Mean Physical Functioning (SD): 89.06 (12.00) Mean Fatigue score (SD): 25.45 (17.96) Mean Pain score (SD): 9.11 (18.35) Mean Dyspnea score (SD): 13.94 (19.66)
		CG (56)	–	Usual care, no postoperative intervention	HRQoL: Mean Summary score (SD): 84.15 (13.17) Mean Global Health (SD): 75.06 (16.02) Mean Physical Functioning (SD): 84.72 (12.06) Mean Fatigue score (SD): 26.51 (18.08) Mean Pain score (SD): 13.34 (18.50) Mean Dyspnea score (SD): 22.33 (19.78)

*CDC* Clavien–Dindo classification, *CG* control group, *CI* confidence interval, *CPAP* continuous positive airway pressure, *ERAS* enhanced recovery after surgery, *HRQoL* health-related quality of life, *ICU* intensive care unit, *IG* intervention group, *IMT* inspiratory muscle training, *IQR* interquartile range, *IS* incentive spirometer, *LL* lower limbs, *LOS* length of hospital stay, *NACT* neoadjuvant chemotherapy, *NRS* non-randomized controlled study, *NS* not specified, *PEP* positive expiratory pressure, *postop* postoperative, *PPCs* postoperative pulmonary complications, *PSM* propensity score matching, *RCT* randomized controlled trial, *SD* standard deviation, *UL* upper limb, *6MWT* 6-min walking test

Finally, six RCTs^38,40–44^ and eight cohort studies^2,46–49,51–53^ assessing prehabilitation, and five RCTs^54–56,58,59^ and five cohort studies^61,62,65,67,68^ assessing peri- or postoperative physiotherapy, were included in the meta-analysis. Included and excluded studies are presented in ES Table S6 for each analysis.

### Functional Exercise Capacity

Due to heterogeneity of outcome measures, a meta-analysis was performed for studies assessing exercise capacity following prehabilitation using the 6MWT. For the final analysis, two RCTs^40,42^ and one cohort^51^ were included (ES Fig. S1). Change in functional capacity was reported from baseline to the postoperative period, with random-effects analysis showing no difference in the mean 6MWT between the two groups (pooled mean difference 26.70, 95% confidence interval [CI] − 73.10 to 126.49; *p* = 0.60).

### Incidence of Pneumonia

**Fig. 2:**
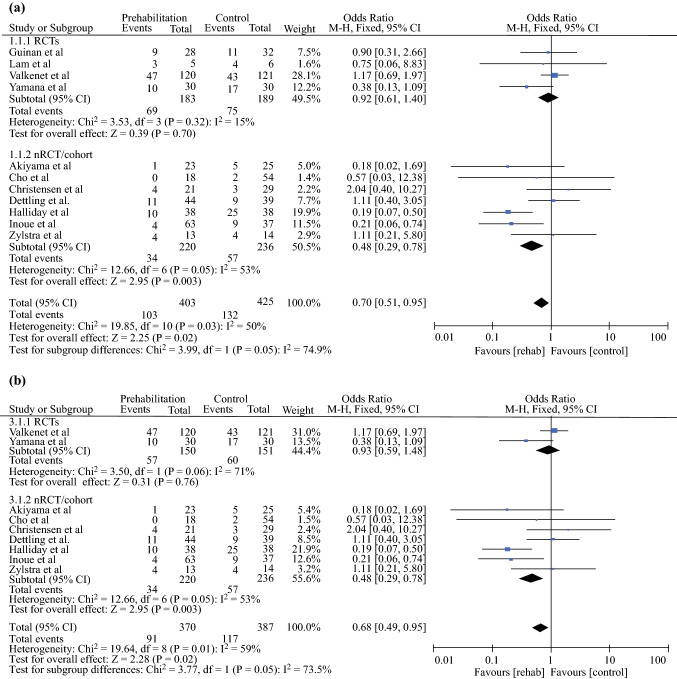

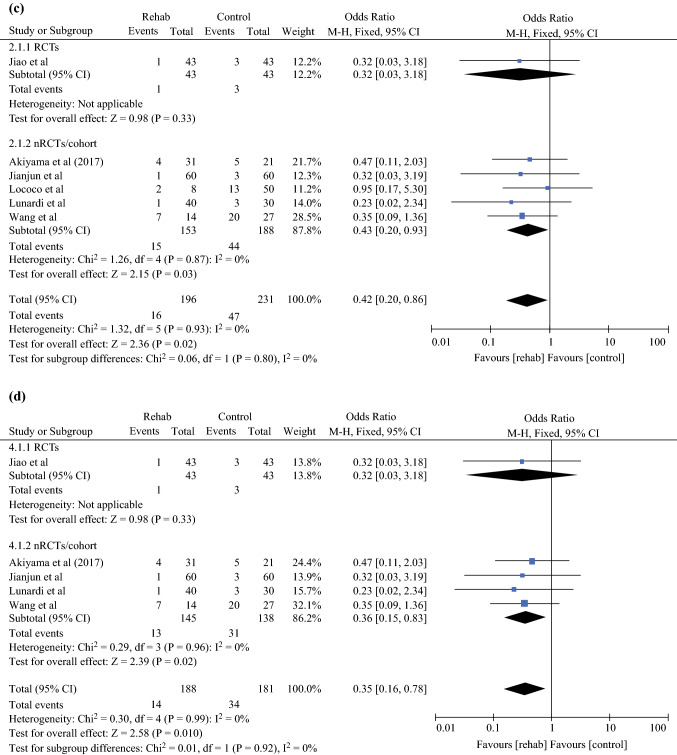
**a** Effect of prehabilitation on the incidence of pneumonia. **b** Effect of prehabilitation on the incidence of pneumonia, excluding the combined incidence of pneumonia and other PPCs. **c** Effect of peri- or postoperative rehabilitation on the incidence of pneumonia. **d** Effect of peri- or postoperative rehabilitation on the incidence of pneumonia, excluding the combined incidence of pneumonia and other PPCs. *PPCs* postoperative pulmonary complications, *M–H* Mantel–Haenszel, *CI* confidence interval, *RCTs* randomized controlled trials, *nRCT* non-randomized controlled trials, *df* degrees of freedom

A meta-analysis was performed for all studies assessing the incidence of pneumonia following prehabilitation (Fig. 2a). Fixed-effects analysis demonstrated a significant difference between the two groups, with a lower incidence of pneumonia in patients receiving prehabilitation (pooled odds ratio [OR] 0.70, 95% CI 0.51–0.95; *p* = 0.02). Further analysis was performed to assess the incidence of pneumonia, with the exclusion of studies providing the combined incidence of pneumonia and other PPCs (Fig. 2b). In agreement with previous findings, fixed-effects analysis showed a significantly lower incidence of pneumonia in patients receiving prehabilitation (pooled OR 0.68, 95% CI 0.49–0.95; *p* = 0.02).

For peri- or postoperative rehabilitation, a meta-analysis was performed for all studies assessing the incidence of pneumonia (Fig. 2c), with fixed-effects analysis demonstrating a significant difference between the two groups, with a lower incidence of pneumonia in the rehabilitation group (pooled OR 0.42, 95% CI 0.20–0.86; *p* = 0.02). Further analysis was performed to assess the incidence of pneumonia, excluding the studies providing the combined incidence of pneumonia and other PPCs (Fig. 2d). Similarly, fixed-effects analysis demonstrated a significantly lower incidence of pneumonia in the rehabilitation group (pooled OR 0.35, 95% CI 0.16–0.78; *p* = 0.01).

### Incidence of Other Postoperative Pulmonary Complications (PPCs)

A meta-analysis was performed for all studies assessing the incidence of other PPCs. Fixed-effects analyses showed no significant difference between the intervention and control groups following either prehabilitation (ES Fig. S2a; pooled OR 0.73, 95% CI 0.51–1.05; *p* = 0.09) or rehabilitation (ES Fig. S2c; pooled OR 1.18, 95% CI 0.60–2.32; *p* = 0.63).

For further analysis, articles that provided the combined incidence of other PPCs and pneumonia were excluded, thus only including studies that reported the incidence of other PPCs. In agreement with earlier findings, fixed-effects analyses showed no difference between the intervention and control groups for the incidence of other PPCs following prehabilitation (ES Fig. S2b; pooled OR 0.71, 95% CI 0.48–1.05; *p* = 0.09) or rehabilitation (ES Fig. S2d; pooled OR 1.23, 95% CI 0.59–2.58; *p* = 0.58).

### Postoperative Morbidity

**Fig. 3:**
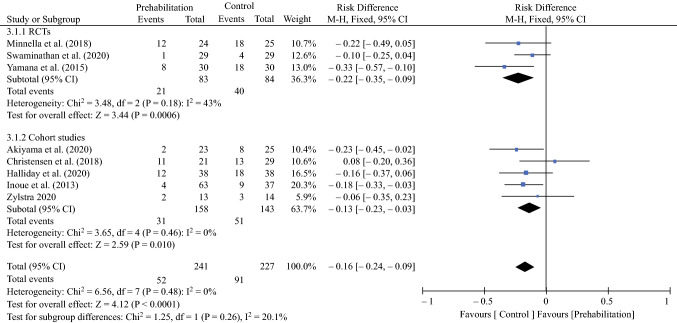
Effect of prehabilitation on the incidence of postoperative morbidity (Clavien–Dindo grade II or higher). *M–H* Mantel–Haenszel, *CI* confidence interval, *RCTs* randomized controlled trials, *df* degrees of freedom

Fixed-effects analysis showed fewer complications in the prehabilitation group compared with the control group (risk difference − 0.16, 95% CI − 0.24 to − 0.09; *p* < 0.0001) (Fig. 3).

### Mortality

Fixed-effects analysis was performed and showed no difference in the in-hospital mortality rates between the two groups (pooled OR 0.97, 95% CI 0.31–3.03; *p* = 0.95) (ES Fig. S3).

### Length of Hospital Stay

**Fig. 4:**
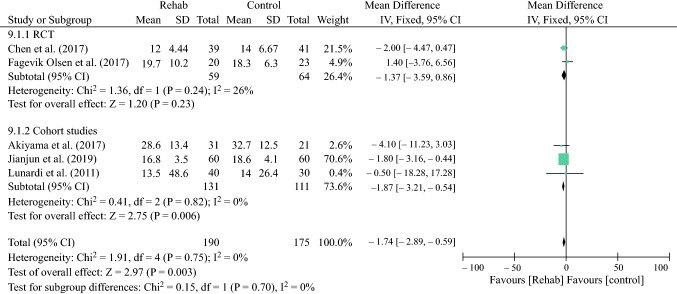
Effect of peri- or postoperative rehabilitation on the LOS. *LOS* length of hospital stay, *SD* standard deviation, *IV* inverse variance, *CI* confidence interval, *RCT* randomized controlled trial, *df* degrees of freedom

Given the high degree of statistical heterogeneity between studies, random-effects analysis was performed and showed no difference between the two groups (mean difference − 0.44, 95% CI − 1.69 to 0.82; *p* = 0.50) after prehabilitation (ES Fig. S4). There was no evidence of statistical heterogeneity between studies reporting the LOS after peri- or postoperative rehabilitation, therefore fixed-effects analysis showed shorter hospital stay in the rehabilitation group (mean difference − 1.74, 95% CI − 2.89 to − 0.59; *p* = 0.003) (Fig. 4).

### Health-Related Quality of Life

**Fig. 5:**
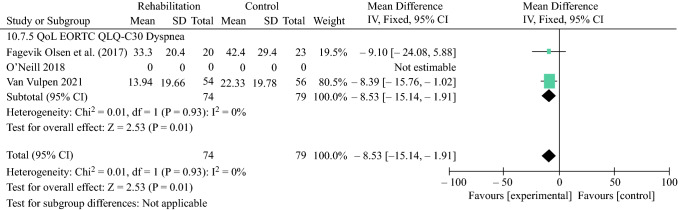
Effect of peri- or postoperative rehabilitation on the EORTC QLQ-C30 Dyspnea. *EORTC* European Organisation for Research and Treatment of Cancer, *SD* standard deviation, *IV* inverse variance, *CI* confidence interval, *df* degrees of freedom, *QoL* quality of life

**Fig. 6:**
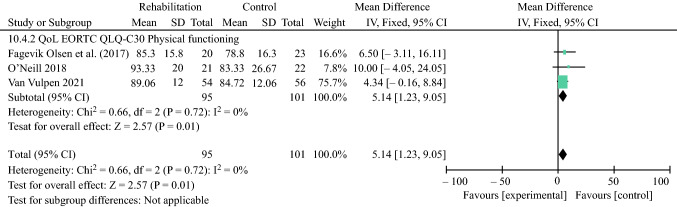
Effect of peri- or postoperative rehabilitation on the EORTC QLQ-C30 Physical Functioning. *EORTC* European Organisation for Research and Treatment of Cancer, *SD* standard deviation, *IV* inverse variance, *CI* confidence interval, *df* degrees of freedom, *QoL* quality of life

A total of three RCTs^55,58,59^ were included in the final analysis. The 3-month outcomes were better after rehabilitation for dyspnea (mean difference − 8.53, 95% CI − 15.14 to 1.91) (Fig. 5) and physical functioning (mean difference 5.14, 95% CI 1.23–9.05) (Fig. 6), while no significant difference was observed for the EORTC QLQ-C30 summary score (ES Fig. S5), global health (ES Fig. S6), fatigue (ES Fig. S7) and pain (ES Fig. S8) between the intervention and control groups.

### Methodological Quality of the Included Studies

A summary of the risk of bias of the included RCTs.^35^ Overall, some concerns were present for the risk of bias assessment of the included RCTs. There were no studies that were considered as high risk of bias. The majority of the included RCTs applied an adequate randomization process with allocation concealment. Half of the studies did not report the method of missing data handling, and, for most studies, there was either inadequate blinding of outcome assessment or no information provided. An evaluation of the cohort studies included in the meta-analysis for the risk of bias is shown in ES Table S7. All but one cohort study received a score of least 6, while seven cohort studies were awarded 7 stars or higher, indicating overall good quality.

## Discussion

In this meta-analysis, lower incidence of pneumonia and postoperative morbidity was observed in patients undergoing prehabilitation, however no significant differences were found for other outcomes. Peri- or postoperative rehabilitation resulted in a lower incidence of pneumonia, a shorter LOS and better HRQoL scores for dyspnea and physical functioning, while no effect was observed for the incidence of other PPCs, QoL summary score, global health status, fatigue, and pain scores.

Enhanced recovery after surgery (ERAS) is a multimodal approach aimed at promoting early recovery in patients undergoing major surgery, and commonly includes a physiotherapy component or early mobilization.^70,71^ These programs have been shown to reduce the risk for complications and decreased the LOS in colorectal surgery.^72^ Prehabilitation also consists of multiple components, such as preoperative exercise intervention, and nutritional and psychological support. Prehabilitation initially comprised of preoperative exercise training, which was developed to improve functional capacity.^73,74^ Nutritional support was subsequently implemented to optimize metabolic reserve preoperatively in order to adequately compensate for the catabolic response following surgery.^75,76^ Similar to findings observed in studies assessing ERAS programs, prehabilitation was shown to improve outcomes in patients undergoing major abdominal or thoracic surgery, with an increase in functional capacity,^77,78^ reduction in complication rates, and shortening in the LOS.^79^

Given the clinical importance of pulmonary complications, the incidence of PPCs was also assessed in this meta-analysis. Both prehabilitation and peri- or postoperative rehabilitation have been shown to reduce the risk for postoperative pneumonia, while no differences were observed for other PPC rates between patients receiving intervention and the control group. Interestingly, the magnitude of improvements in pneumonia and LOS were greater with peri- or postoperative rehabilitation than prehabilitation. There are several possible explanations for this, including the proximity in timing of the intervention to the measurement of the outcome, and, second, compliance with the intervention, with peri- and postoperative rehabilitation performed in the hospital setting. However, differences were observed between the RCTs and cohort studies, since RCTs showed no difference in the incidence of pneumonia following prehabilitation and rehabilitation. This could be explained by the low number of RCTs assessing prehabilitation and peri- or postoperative rehabilitation. For the analysis assessing the incidence of pneumonia after prehabilitation, the study by Valkenet et al.^44^ was given a greater weight than other RCTs, thus determining the outcome. The authors implemented inspiratory muscle training using an inspiratory loading device. The intervention was however home-based and only half of the participants (54.2%) trained at least 80% of the planned sessions and 28% of the participants trained at least 80% of the sessions at the prescribed intensity. The study by Jiao et al.^54^ was the only RCT included in the analysis that assessed the impact of peri- or postoperative rehabilitation. The physiotherapy intervention consisted of preoperative deep breathing exercises with balloons and abdominal and pursed-lips breathing training followed by postoperative assisted sputum elimination. The exercises were commenced in the preoperative setting and continued in the immediate postoperative period throughout the hospital admission, with no specification of the duration and intensity of the training. Interestingly, both Valkenet et al.^44^ and Jiao et al.^54^ reported significantly improved respiratory function in the intervention group compared with the control group, with a higher increase in respiratory muscle strength (maximal inspiratory muscle strength and inspiratory muscle endurance capacity),^44^ and higher respiratory function indices (forced vital capacity and peak expiratory flow),^54^ respectively.

HRQoL status was assessed in patients undergoing peri- or postoperative rehabilitation at 3 months following surgery. All three RCTs^55,58,59^ implemented a 12-week rehabilitation program, two^58,59^ of which implemented a program consisting of aerobic and resistance training, while the third study^55^ included breathing exercises, strength training, and optimization of thoracic spine mobility. At 2 years after surgery, Fagevik Olsén et al.^80^ found clinically significant worse QoL scores for dyspnea, fatigue, diarrhea, and appetite loss. Another study presented similar findings, showing persistently worse QoL scores for physical functioning and dyspnea at 3 years after surgery.^81^ Moreover, pain around chest scars and reduced energy or activity tolerance were associated with long-term poor HRQoL.^82^ Lastly, the LOS did not differ in the prehabilitation group, while peri- or postoperative exercise intervention resulted into a significantly shorter LOS. Rehabilitation may therefore aid in earlier in-hospital recovery.

There are several limitations present in this meta-analysis. Due to the limited number of RCTs conducted, cohort studies were included in the analysis. For most RCTs, allocation was concealed, there was a low dropout rate following randomization, and the intervention and control groups were similar at baseline for most trials. However, several studies did not report handling of missing data and the reasons for dropout, and the majority of studies reported no blinding of outcome assessment. Several cohort studies used historical controls as a comparison group, consisting of patients who had undergone surgery before physiotherapy implementation. No subgroup analysis could be performed for the type of surgery, including the surgical approach (minimally invasive surgery [MIS] or open surgery), as some studies only provided the number of patients undergoing open surgery or MIS, while the number of patients undergoing either esophagectomy or gastrectomy within these subcohorts was not specified.

The short-term outcomes were assessed by well-defined measures, such as the CDC for surgical complications, and by reporting the mortality rates. The criteria for diagnosis of pneumonia were well-described in four studies,^2,38,44,52^ which were based on leukocyte count, presence of fever, sputum and chest X-Ray findings. However, the remaining studies reported the incidence of pneumonia or PPCs only. Perioperative rehabilitation was implemented in only a small number of studies, while postoperative rehabilitation was commonly commenced after discharge or in long-term survivors. Although a Clavien–Dindo score of 3 or higher is commonly considered for clinically complications, in this meta-analysis a cut-off at a score of 2 was used to assess the incidence of postoperative morbidity in order to include the incidence of pneumonia, classified as complications requiring pharmacological treatment (Clavien–Dindo score 2).

Patients with esophageal and gastric cancer commonly experience ongoing malnutrition after surgery and often report poor long-term physical functioning and ongoing respiratory symptoms.^81,83–85^ Physical activity levels were measured by obtaining the step count with an accelerometer. A total of three studies have reported physical activity, two of which assessed this outcome following prehabilitation,^41,42^ and only one study assessed this after rehabilitation in long-term survivors.^58^ The respiratory function was evaluated by estimating the respiratory volumes and by measuring the respiratory muscle pressure and endurance. A total of eight studies (four in the preoperative period^42,44,45,52^ and four in the peri- or postoperative period^54,55,60,68^) reported the respiratory function, with only one study^64^ assessing the long-term outcomes. No meta-analysis could be performed due to different timing of measurements and the use of different parameters. This suggests that there is a lack of research assessing functional data in long-term survivorship undergoing rehabilitation. There was no standardized regimen as exercise interventions differed in timing and duration, and were either home-based or in-hospital, with or without supervision. The Borg scale was used to estimate the intensity of the intervention for a large number of studies. However, no standardized measure was available to compare all regimens. To date, there is an evident paucity of research comparing the impact the different components, intensity, and setting of physiotherapy in patients who have undergone esophagectomy or gastrectomy.

Finally, adherence was reported in the majority of studies included in the meta-analysis. For most studies, adherence was monitored by a physiotherapist, while a few studies implemented objective measures such as heart rate monitors, which could possibly improve the objectivity of adherence reporting.

## Conclusion

The findings of this meta-analysis showed that implementation of exercise intervention may be beneficial in both the preoperative and peri- or postoperative periods. The next steps of the investigation are to identify which components, or pre-habilitation and peri- and postoperative rehabilitation, have the greatest impact on the clinical outcomes. Furthermore, clearly the Achilles heel to prehabilitation and rehabilitation is patient compliance; more research is needed to understand the human factors and patient barriers around complications to these regimens, to ensure the long-term clinical effectiveness.

## Supplementary Information

Below is the link to the electronic supplementary material.

Supplementary file 1 (DOCX 2888 KB)

Supplementary file 2 (DOCX 184 KB)
