# Supplementary material for: Physiotherapy Regimens in Esophagectomy and Gastrectomy: a Systematic Review and Meta-Analysis
Source: Ann Surg Oncol. 2021 Dec 27;29(5):3148–67. doi: 10.1245/s10434-021-11122-7 (PMC8990957; doi:10.1245/s10434-021-11122-7)

Supplemental figure 1: The effect of prehabilitation on functional exercise capacity


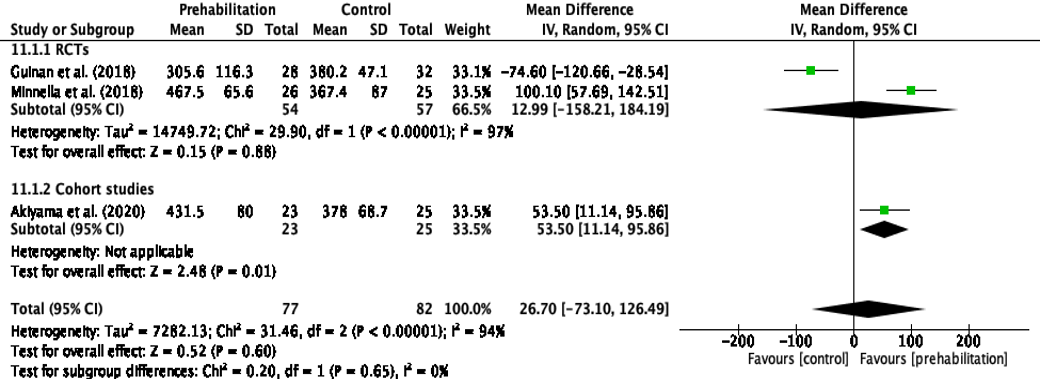


Supplemental figure 2a: The effect of prehabilitation on the incidence of other PPC


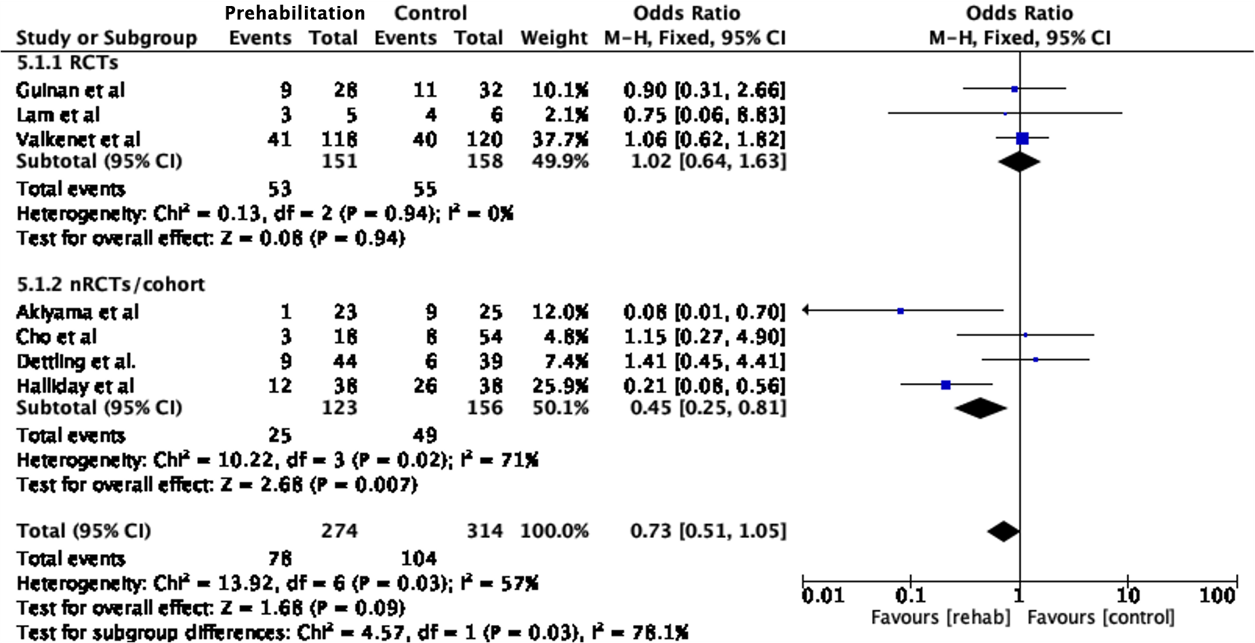


Supplemental figure 2b: The effect of prehabilitation on the incidence of other PPC, excluding the combined incidence of pneumonia and other PPC


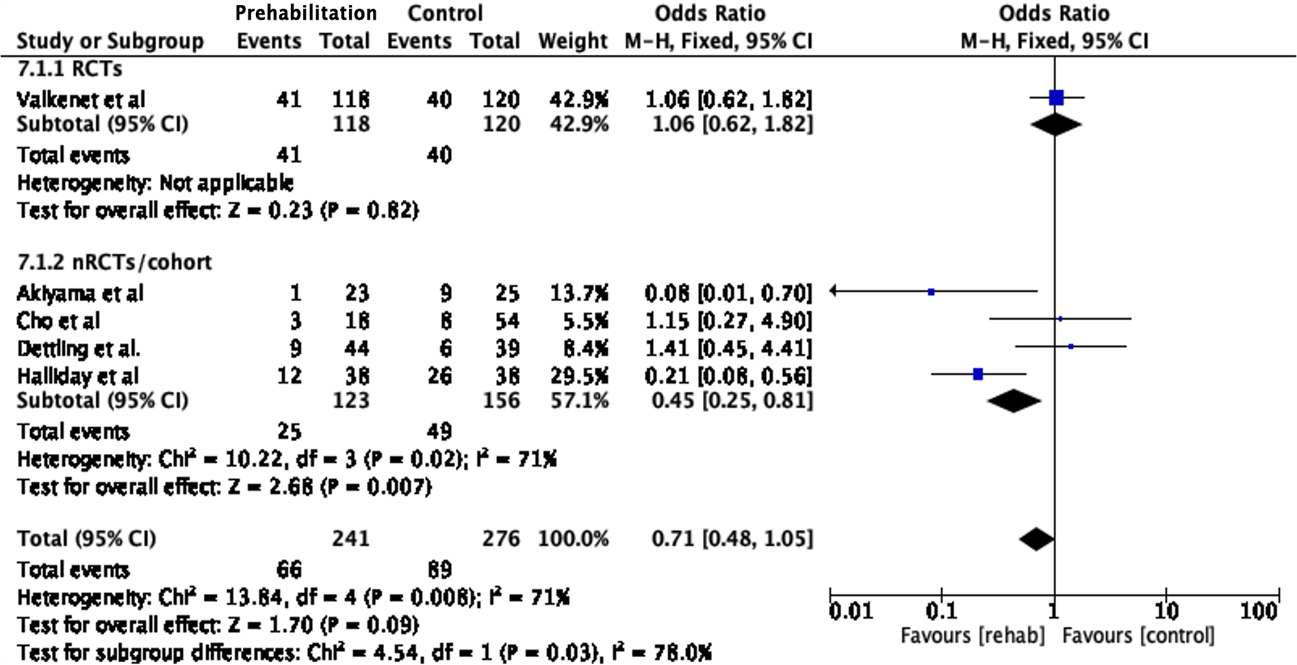


Supplemental figure 2c: The effect of peri- or postoperative rehabilitation on the incidence of other PPC


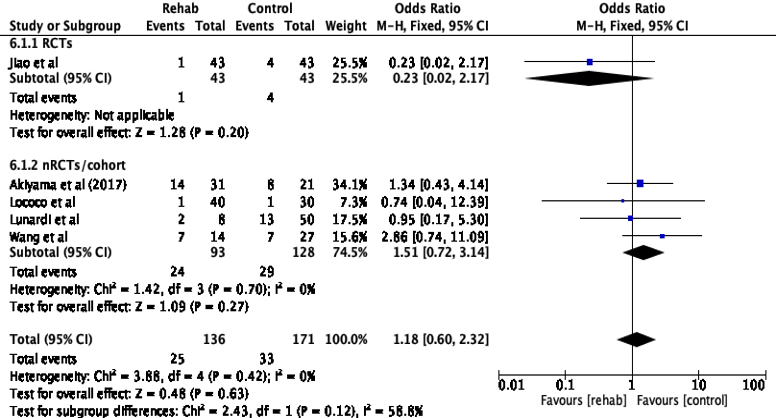


Supplemental figure 2d: The effect of peri- or postoperative rehabilitation on the incidence of other PPC, excluding the combined incidence of pneumonia and other PPC


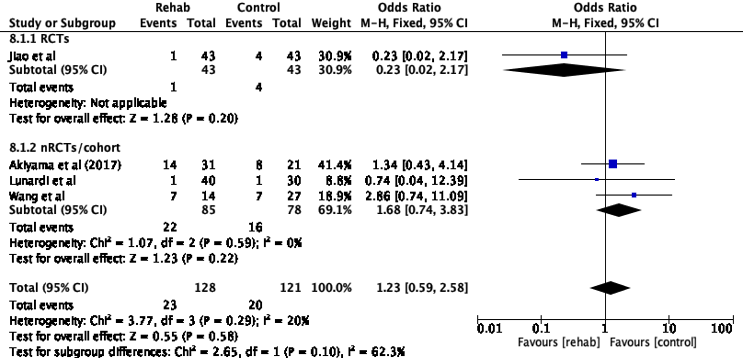


Supplemental figure 3: The effect of prehabilitation on in-hospital mortality


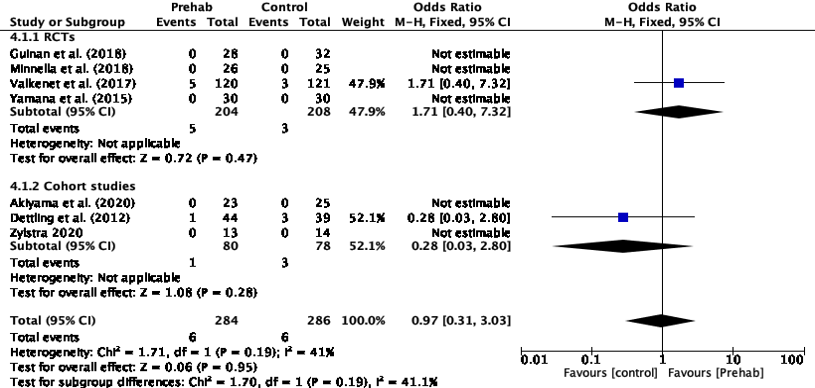


Supplemental figure 4: The effect of prehabilitation on the LOS


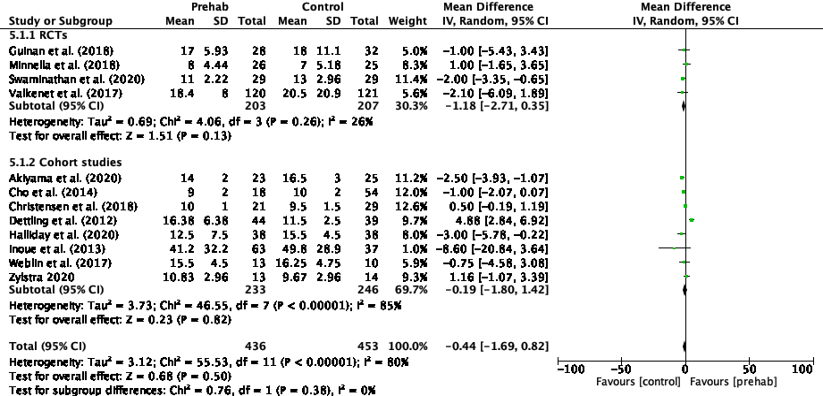


Supplemental figure 5: The effect of peri- or postoperative rehabilitation on the EORTC QLQ-C30 Summary scores


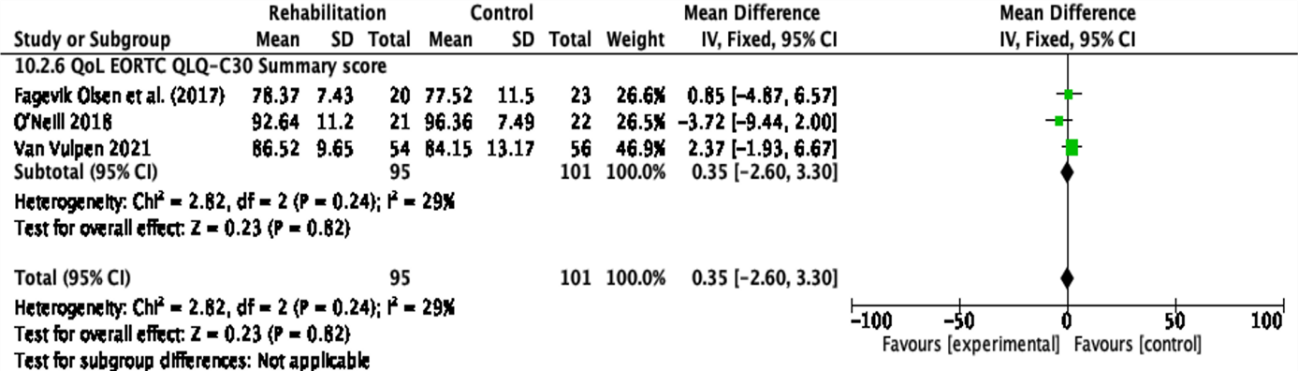


Supplemental figure 6: The effect of peri- or postoperative rehabilitation on the EORTC QLQ-C30 Global HRQoL


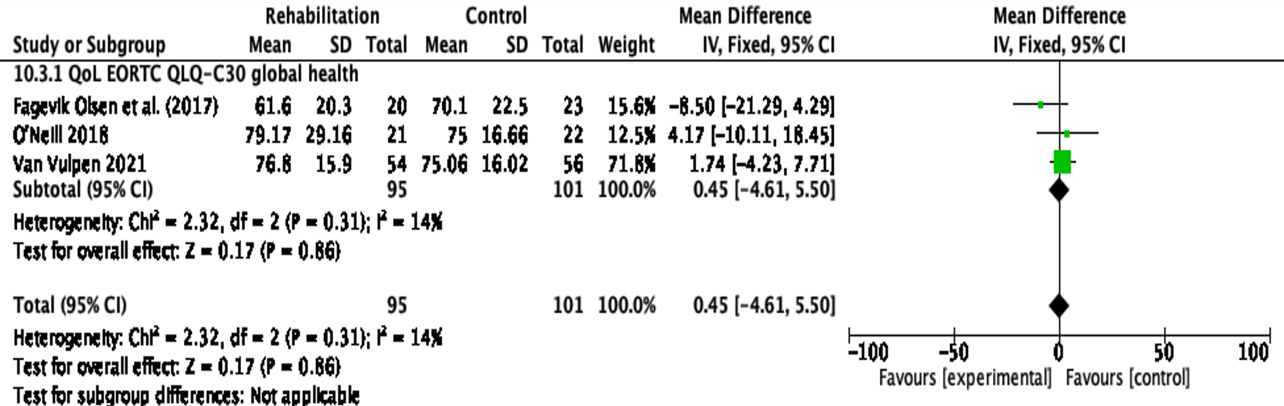


Supplemental figure 7: The effect of peri- or postoperative rehabilitation on the EORTC QLQ-C30 Fatigue


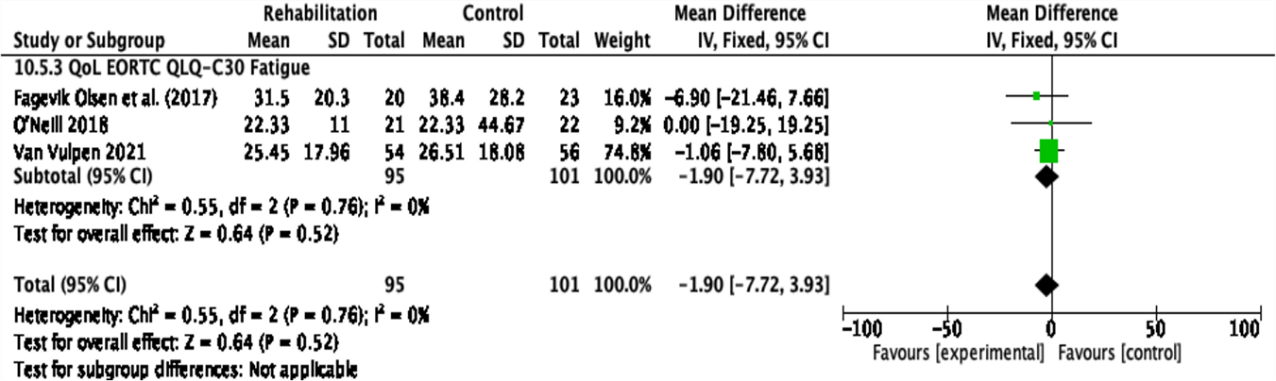


Supplemental figure 8: The effect of peri- or postoperative rehabilitation on the EORTC QLQ-C30 Pain


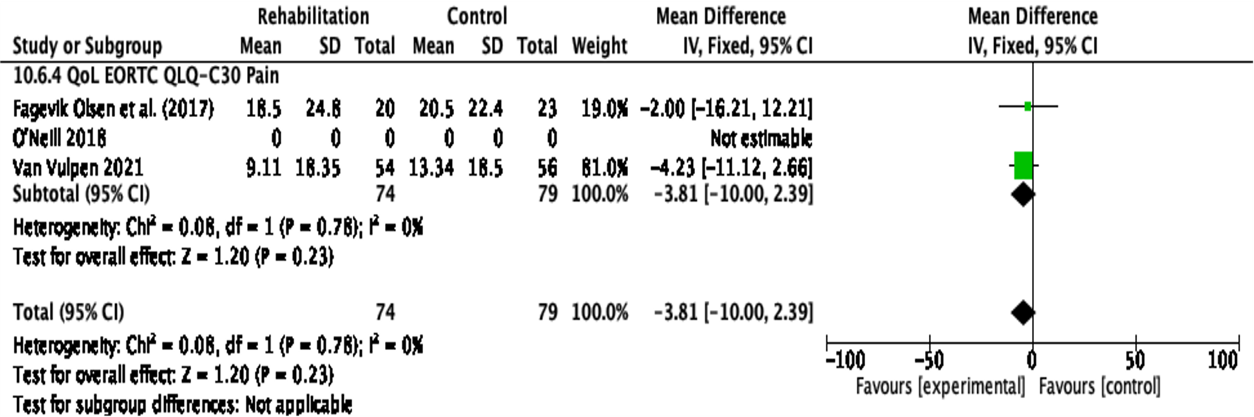


Supplemental figure S9: Cochrane Risk-of-Bias tool for randomized trials version 2 (RoB-2)


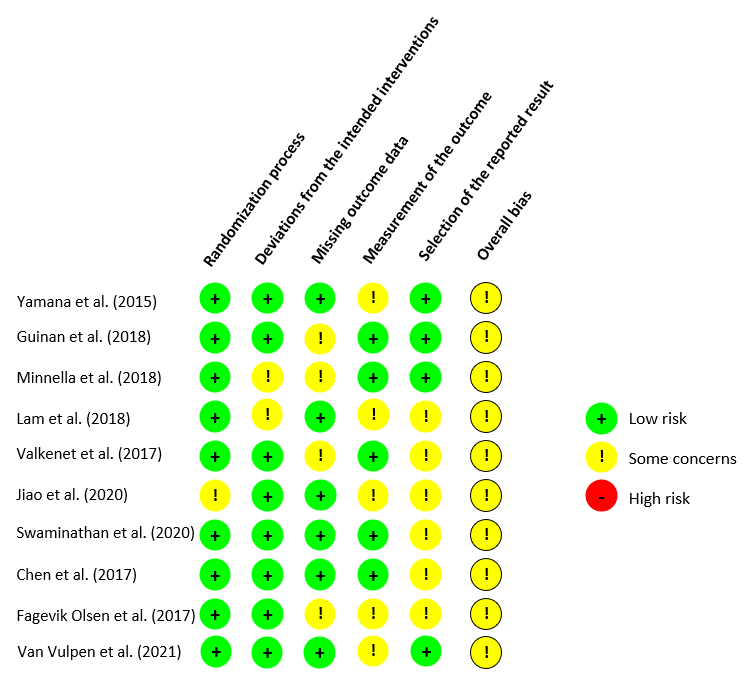

Supplement: Supplementary file 1 — (DOCX 2888 KB) [file 10434_2021_11122_MOESM1_ESM.docx]
