# Supplementary material for: Physiotherapy Regimens in Esophagectomy and Gastrectomy: a Systematic Review and Meta-Analysis
Source: Ann Surg Oncol. 2021 Dec 27;29(5):3148–67. doi: 10.1245/s10434-021-11122-7 (PMC8990957; doi:10.1245/s10434-021-11122-7)
Supplement: Supplementary file 2 — (DOCX 184 KB) [file 10434_2021_11122_MOESM2_ESM.docx]

**SUPPLEMENTAL TABLES**

Supplementary table S1: search strategy

| **Database** | **Search** | **Query** | **Items found** |
| --- | --- | --- | --- |
| Embase | #1 | physiotherapy.mp. OR mobili?ation.mp. OR physical therap*.mp. OR physical treatment.mp. OR rehabilitat*.mp. OR physical activit*.mp. OR exercis*.mp. OR kinesi*therap*.mp. OR incentive spiromet*.mp. OR respiratory muscle training.mp. OR inspiratory muscle training.mp. OR respiratory therap*.mp. OR breathing technique*.mp. OR positive expiratory pressure.mp. OR positive pressure expiration.mp. OR positive pressure breathing.mp. OR expansion breathing OR expansion exercise* OR cough* exercise* OR ventilat* muscle training OR exp physiotherapy practice/ OR exp physiotherapy/ OR exp home physiotherapy/ OR exp rehabilitation patient/ OR exp pulmonary rehabilitation/ OR exp rehabilitation center/ OR exp rehabilitation nursing/ OR exp community based rehabilitation/ OR exp home rehabilitation/ OR exp rehabilitation/ OR exp rehabilitation care/ OR exp rehabilitation medicine/ OR exp mobilization/ OR exp muscle exercise/ OR exp exercise/ OR exp exercise recovery/ OR exp exercise tolerance/ OR exp treadmill exercise/ OR exp breathing exercise/ OR exp aerobic exercise/ OR exp exercise intensity/ OR exp kinesiotherapy/ OR exp muscle training/ | 1507396 |
|  | #2 | gastrectomy.mp. OR exp gastrectomy Billroth I/ OR exp partial gastrectomy/ OR exp gastrectomy/ OR exp gastrectomy Billroth II/ OR exp stomach cancer/ OR exp stomach carcinoma/ OR exp stomach adenocarcinoma/ OR [(gastric.mp. OR stomach.mp.) AND ((cancer.mp. OR carcinoma.mp. OR neoplasm*.mp. OR tumo*r.mp. OR malign*.mp. OR adenocarcino*.mp. OR squamous.mp.)] OR ivor lewis.mp. OR mckeown.mp. OR esophagectomy.mp. OR oesophagectomy.mp. OR exp esophagus resection/ OR exp esophagus cancer/ OR exp esophagus carcinoma/ exp esophageal adenocarcinoma/ OR exp esophageal squamous cell carcinoma/ OR [(esophagus.mp. OR oesophagus.mp. OR esophageal.mp. OR oesophageal.mp.) AND (cancer.mp. OR carcinoma.mp. OR neoplasm*.mp. OR tumo*r.mp. OR malign*.mp. OR adenocarcino*.mp. OR squamous.mp.)] OR [(esophagus.mp. OR oesophagus.mp. OR esophageal.mp. OR oesophageal.mp.) AND resection.mp.] | 408563 |
|  | #3 | #1 AND #2 | 7290 |
|  | #4 | Limit #3 to yr=”1990-Current” | 6413 |
| Medline | #1 | exp Exercise Therapy/ OR exp Physical Therapy Modalities/ OR physiotherapy.mp. OR physical therap*.mp. OR mobili?ation.mp. OR physical treatment.mp. OR exp Rehabilitation/ OR rehabilitat*.mp. OR exp Rehabilitation Centers/ OR physical activit*.mp. OR exp Exercise/ OR exercis*.mp. OR kinesi*therap*.mp. OR exp Respiratory Therapy/ OR incentive spirometry.mp. OR exp Breathing Exercises/ OR incentive spirometer.mp. OR respiratory muscle training.mp. OR inspiratory muscle training.mp. OR respiratory therap*.mp. OR breathing technique*.mp. OR positive expiratory pressure.mp. OR positive pressure expiration.mp. OR positive pressure breathing.mp. OR exp "Physical and Rehabilitation Medicine"/ OR exp Rehabilitation Nursing/ OR exp Hospitals, Rehabilitation/ OR exp Physical Exertion/ OR expansion breathing OR expansion exercise* OR cough* exercise* OR ventilatory muscle training | 1180353 |
|  | #2 | ivor lewis.mp. OR mckeown.mp. exp Esophageal Neoplasms/ OR exp Esophageal Squamous Cell Carcinoma/ OR esophagectomy.mp. OR exp Esophagectomy/ OR oesophagectomy.mp.OR [resection.mp. AND (esophagus.mp. OR oesophagus.mp. OR esophageal.mp. OR oesophageal.mp.)] OR [(esophagus.mp. OR oesophagus.mp. OR esophageal.mp. OR oesophageal.mp.) AND (cancer.mp. OR tumo*r.mp. OR neoplasm*.mp. OR malign*.mp. OR adenocarcino*.mp. OR squamous.mp.)] OR gastrectomy.mp. OR exp Gastrectomy/ OR exp Stomach Neoplasms/ OR [(cancer.mp. OR tumo*r.mp. OR neoplasm*.mp. OR malign*.mp. OR adenocarcino*.mp. OR squamous.mp.) AND (stomach.mp. OR gastric.mp.)] | 249112 |
|  | #3 | #1 AND #2 | 3290 |
|  | #4 | Limit #3 to yr=”1990-Current” | 2722 |
| Cochrane | #1 | MeSH descriptor: [Physical Therapy Modalities] explode all trees OR MeSH descriptor: [Rehabilitation] explode all trees OR MeSH descriptor: [Physical Therapy Specialty] explode all trees OR MeSH descriptor: [Rehabilitation Nursing] explode all trees OR (physiotherapy OR mobilisation OR mobilization OR physical therap* OR physical treatment OR rehabilitat* OR exercis* OR kinesi*therap* OR incentive spiromet* OR respiratory muscle training OR breathing technique* OR inspiratory muscle training OR respiratory therap* OR positive pressure expiration OR positive expiratory pressure OR positive pressure breathing OR expansion breathing OR expansion exercise* OR cough* exercise OR ventilatory muscle training):ti,ab,kw | 254277 |
|  | #2 | MeSH descriptor: [Esophagectomy] explode all trees OR MeSH descriptor: [Esophageal Neoplasms] explode all trees OR (oesophagectomy OR esophagectomy OR esophagus resection OR oesophagus resection OR esophageal resection OR oesophageal resection OR ivor lewis OR mckeown OR ):ti,ab,kw MeSH descriptor: [Stomach Neoplasms] explode all trees OR [(oesophagus OR esophagus OR oesophageal OR esophageal):ti,ab,kw AND (cancer OR carcinoma OR neoplasm* OR tumo*r OR malign* OR adenocarcino* OR squamous):ti,ab,kw] OR [(gastric OR stomach):ti,ab,kw AND (cancer OR carcinoma OR neoplasm* OR tumo*r OR malign* OR adenocarcino* OR squamous):ti,ab,k] | 16487 |
|  | #3 | #1 AND #2 | 1079 |
|  | #4 | Limit #3 to yr=”1990-Current” | 1079 |
| CINAHL | #1 | physiotherapy OR physical therap* OR mobili?ation OR physical treatment OR rehabilitat* OR physical activit* OR exercis* OR kinesi*therap* OR incentive spiromet* OR respiratory muscle* training OR inspiratory muscle* training OR respiratory therap* OR breathing technique* OR positive expiratory pressure OR positive pressure expiration OR positive pressure breathing OR exercise recovery OR kinesiotherapy OR kinesitherapy OR muscle* exercise* OR expansion breathing OR expansion exercise* OR cough* exercise* OR ventilatory muscle* training OR (MH “Activities of Daily Living+”) OR (MH "Early Ambulation") OR (MH "Home Rehabilitation+") OR (MH "Physical Therapy+") OR (MH "Rehabilitation, Pulmonary+") OR (MH "Chest Physical Therapy+") OR (MH "Therapeutic Exercise+") OR (MH "Respiratory Therapy+") OR (MH "Recovery, Exercise") | 630245 |
|  | #2 | Gastrectomy OR oesophagectomy OR esophagectomy OR ivor lewis OR mckeown OR [(gastric OR stomach OR esophagus OR oesophagus OR esophageal OR oesophageal ) AND (cancer OR tumor OR tumour OR carcinoma OR neoplasm* OR malign*)] OR (MH "Gastrectomy+" OR MH "Stomach Neoplasms") OR (MH "Esophageal Neoplasms") | 31365 |
|  | #3 | #1 AND #2 | 897 |
|  | #4 | #3 AND ‘Published Date: 19900101-20201231” | 897 |
| PEDro | #1 | *esophagectomy OR *esophagus OR *esophageal OR ivor lewis OR gastrectomy OR stomach OR gastric | 169 |
|  | #2 | #1 AND “Published since 1990” | 162 |

Supplemental table S2: PRISMA 2020 checklist

| **Section and Topic** | **Item #** | **Checklist item** | **Location where item is reported** |
| --- | --- | --- | --- |
| **TITLE** | | |  |
| Title | 1 | Identify the report as a systematic review. | Title page |
| **ABSTRACT** | | |  |
| Abstract | 2 | See the PRISMA 2020 for Abstracts checklist. | Abstract |
| **INTRODUCTION** | | |  |
| Rationale | 3 | Describe the rationale for the review in the context of existing knowledge. | P4 |
| Objectives | 4 | Provide an explicit statement of the objective(s) or question(s) the review addresses. | P4, 5 |
| **METHODS** | | |  |
| Eligibility criteria | 5 | Specify the inclusion and exclusion criteria for the review and how studies were grouped for the syntheses. | P6, 7 |
| Information sources | 6 | Specify all databases, registers, websites, organisations, reference lists and other sources searched or consulted to identify studies. Specify the date when each source was last searched or consulted. | P6 |
| Search strategy | 7 | Present the full search strategies for all databases, registers and websites, including any filters and limits used. | P6, suppl. table 1 |
| Selection process | 8 | Specify the methods used to decide whether a study met the inclusion criteria of the review, including how many reviewers screened each record and each report retrieved, whether they worked independently, and if applicable, details of automation tools used in the process. | P6 |
| Data collection process | 9 | Specify the methods used to collect data from reports, including how many reviewers collected data from each report, whether they worked independently, any processes for obtaining or confirming data from study investigators, and if applicable, details of automation tools used in the process. | P6 |
| Data items | 10a | List and define all outcomes for which data were sought. Specify whether all results that were compatible with each outcome domain in each study were sought (e.g. for all measures, time points, analyses), and if not, the methods used to decide which results to collect. | P6, 7 |
|  | 10b | List and define all other variables for which data were sought (e.g. participant and intervention characteristics, funding sources). Describe any assumptions made about any missing or unclear information. | P6, 7 |
| Study risk of bias assessment | 11 | Specify the methods used to assess risk of bias in the included studies, including details of the tool(s) used, how many reviewers assessed each study and whether they worked independently, and if applicable, details of automation tools used in the process. | P7, 8 |
| Effect measures | 12 | Specify for each outcome the effect measure(s) (e.g. risk ratio, mean difference) used in the synthesis or presentation of results. | P8 |
| Synthesis methods | 13a | Describe the processes used to decide which studies were eligible for each synthesis (e.g. tabulating the study intervention characteristics and comparing against the planned groups for each synthesis (item #5)). | P8 |
|  | 13b | Describe any methods required to prepare the data for presentation or synthesis, such as handling of missing summary statistics, or data conversions. | N/A |
|  | 13c | Describe any methods used to tabulate or visually display results of individual studies and syntheses. | P8 |
|  | 13d | Describe any methods used to synthesize results and provide a rationale for the choice(s). If meta-analysis was performed, describe the model(s), method(s) to identify the presence and extent of statistical heterogeneity, and software package(s) used. | P8 |
|  | 13e | Describe any methods used to explore possible causes of heterogeneity among study results (e.g. subgroup analysis, meta-regression). | P8 |
|  | 13f | Describe any sensitivity analyses conducted to assess robustness of the synthesized results. | N/A |
| Reporting bias assessment | 14 | Describe any methods used to assess risk of bias due to missing results in a synthesis (arising from reporting biases). | P8 |
| Certainty assessment | 15 | Describe any methods used to assess certainty (or confidence) in the body of evidence for an outcome. | N/A |
| **RESULTS** | | |  |
| Study selection | 16a | Describe the results of the search and selection process, from the number of records identified in the search to the number of studies included in the review, ideally using a flow diagram. | P9, figure 1 |
|  | 16b | Cite studies that might appear to meet the inclusion criteria, but which were excluded, and explain why they were excluded. | P9 |
| Study characteristics | 17 | Cite each included study and present its characteristics. | P9-16 |
| Risk of bias in studies | 18 | Present assessments of risk of bias for each included study. | Suppl. table 6 and figure 8 |
| Results of individual studies | 19 | For all outcomes, present, for each study: (a) summary statistics for each group (where appropriate) and (b) an effect estimate and its precision (e.g. confidence/credible interval), ideally using structured tables or plots. | P9-16, suppl. table 3-5 |
| Results of syntheses | 20a | For each synthesis, briefly summarise the characteristics and risk of bias among contributing studies. | P16 |
|  | 20b | Present results of all statistical syntheses conducted. If meta-analysis was done, present for each the summary estimate and its precision (e.g. confidence/credible interval) and measures of statistical heterogeneity. If comparing groups, describe the direction of the effect. | P9-16, suppl. figures 1-7 |
|  | 20c | Present results of all investigations of possible causes of heterogeneity among study results. | P9-16 |
|  | 20d | Present results of all sensitivity analyses conducted to assess the robustness of the synthesized results. | N/A |
| Reporting biases | 21 | Present assessments of risk of bias due to missing results (arising from reporting biases) for each synthesis assessed. | P16 |
| Certainty of evidence | 22 | Present assessments of certainty (or confidence) in the body of evidence for each outcome assessed. | N/A |
| **DISCUSSION** | | |  |
| Discussion | 23a | Provide a general interpretation of the results in the context of other evidence. | P17-21 |
|  | 23b | Discuss any limitations of the evidence included in the review. | P21, 22 |
|  | 23c | Discuss any limitations of the review processes used. | P21, 22 |
|  | 23d | Discuss implications of the results for practice, policy, and future research. | P23 |
| **OTHER INFORMATION** | | |  |
| Registration and protocol | 24a | Provide registration information for the review, including register name and registration number, or state that the review was not registered. | P23 |
|  | 24b | Indicate where the review protocol can be accessed, or state that a protocol was not prepared. | P23 |
|  | 24c | Describe and explain any amendments to information provided at registration or in the protocol. | N/A |
| Support | 25 | Describe sources of financial or non-financial support for the review, and the role of the funders or sponsors in the review. | N/A |
| Competing interests | 26 | Declare any competing interests of review authors. | Title page |
| Availability of data, code and other materials | 27 | Report which of the following are publicly available and where they can be found: template data collection forms; data extracted from included studies; data used for all analyses; analytic code; any other materials used in the review. | Suppl. material |

Supplemental table S3: Summary of study characteristics of all studies

| **Authors (year)** | **Study type** | **Group (n)** | **Participants characteristics** | **Surgery** | **Other treatment modality** |
| --- | --- | --- | --- | --- | --- |
| **PREHABILITATION** | | | | | |
| ***Dettling et al. (2012)**^63^ | NRS | IG (n=44) | Mean age: 65.1 yr old M/F: 33/11 | OE (n=35) MIE (n=9) | NACRT (n=31) |
|  |  | CG (n=39) | Mean age: 66.5 yr old M/F: 29/10 | OE (n=35) MIE (n=4) | NACRT (n=17) |
| ***Inoue et al. (2013)**^9^ | Cohort study | IG (n=63) | Mean age: 67.4 yr old M/F: 53/10 | OE (n=6) MIE (n=57) | NACT (n=43) |
|  |  | CG (n=37) | Mean age: 65 yr old M/F: 34/3 | MIE (n=37) | NACT (n=16) |
| ***Cho et al. (2014)**^60^ | Cohort study | IG (n=18) | Mean age: 63.1 yr old M/F: 18/0 | OG (n=9)  MIG (n=9) | NS |
|  |  | CG (n=54) | Mean age: 66.1 yr old M/F: 51/3 | OG (n=26) MIG (n=28) | NS |
| Van Adrichem et al. (2014)^56^ | RCT | IG1 (n=20) | Mean age: 62.7 yr old  M/F: 15/5 | OE (n=20) | NACRT (n=18) |
|  |  | IG2 (n=19) | Mean age: 61.3 yr old M/F: 14/5 | OE (n=19) | NACRT (n=18) |
| ***Yamana et al. (2015)**^49^ | RCT | IG (n=30) | Mean age: 68.33 yr old M/F: 24/6 | OE (n=7) MIE (n=23) | NACT (n=13) NART (n=3) |
|  |  | CG (n=30) | Mean age: 65.90 yr old M/F: 23/7 | OE (n=9) MIE (n=21) | NACT (n=15) NART (n=5) |
| Xu et al. (2015)^50^ | RCT | IG (n=28) | Mean age: 58.1 yr old M/F: 26/2 | Esophagectomy approach NS | NACRT (n=28) |
|  |  | CG (n=28) | Mean age: 61.1 yr old M/F: 26/2 | Esophagectomy approach NS | NACRT (n=28) |
| ***Weblin et al. (2017)**^59^ | NRS | IG (n= 13) | Mean age: 67 yr old M/F: NS | OE or MIE; OG or MIG | NACT (n=13) |
|  |  | CG (n=10) | Mean age: 63.8 yr old M/F: NS | OE or MIE; OG or MIG | NACT (n=10) |
| ***Valkenet et al. (2017)**^55^ | RCT | IG (n=120) | Mean age: 63.7 yr old M/F: 89/31 | OE (n=44) MIE (n=76) | NACT (n=10) NACRT (n=93) |
|  |  | CG (n=121) | Mean age: 62.7 yr old M/F: 97/24 | OE (n=47) MIE (n=74) | NACT (n=12) NACRT (n=94) |
| ***Christensen et al. (2018)**^57^ | NRS | IG (n= 21) | Mean age: 63.9yr old M/F: 18/3 | OE (n=17) MIE (n=0) HE (n=2) | NACT (n=18) NACRT (n=2) |
|  |  | CG (n= 29 ) | Mean age: 65.5 yr old M/F: 27/2 | OE (n=16) MIE (n=2) HE (n=5) | NACT (n=22) NACRT (n=4) |
| Herrstedt et al. (2018)^61^ | NRS | IG (n= 18 ) | Mean age: 63.8 yr old M/F: 15/3 | NS | NACT (n=16) NACRT (n=2) |
|  |  | CG (n=25 ) | Mean age: 65.4 yr old M/F: 23/2 | NS | NACT (n=21) NACRT (n=4) |
| ***Minnella et al. (2018)**^51^ | RCT | IG (n=26) | Mean age: 67.3 yr old M/F: 18/8 | Open (n=16) or MIS (n=10) Esophagectomy (n=18) Gastrectomy (n=6) | NACT (n=20) |
|  |  | CG (n=25) | Mean age: 68.0 yr old M/F: 20/5 | Open (n=14) or MIS (n=11) Esophagectomy (n=21) Gastrectomy (n=4) | NACT (n=15) |
| ***Guinan et al. (2018)**^53^ | RCT | IG (n=28) | Mean age: 63.07 yr old M/F: 20/8 | OE (n=28) | Perioperative CT (n=6) NACRT (n=16) |
|  |  | CG (n=32) | Mean age: 65.06 yr old M/F: 22/10 | OE (n=32) | Perioperative CT (n=9) NACRT (n=14) |
| ***Lam et al. (2018)**^52^ | RCT, Thesis | IG (n=5) | * mean age: 66.3 yr old * males/females: 4/1 | HE or MIE (n=5) | NACT (n=5) |
|  |  | CG (n=6) | * mean age: 65.4 yr old * males/females: 6/0 | HE or MIE (n=6) | NACT (n=6) |
| ***Akiyama et al. (2020)**^62^ | Cohort Study | IG (n=23) | Mean age: 65.9 yr old M/F: 17/6 | OE (n=1) MIE (n=22) | NACT (n=16) NACRT (n=1) |
|  |  | CG (n=25) | Median age: 65.6 yr old M/F: 21/4 | OE (n=1) MIE (n=24) | NACT (n=10) NACRT (n=1) |
| ***Halliday et al. (2020)**^58^ | Cohort Study | IG (n=38) | Mean age: 69.0 yr old M/F: NS | OE (n=38) | NACT (n=33) |
|  |  | CG (n=38) | Median age: 68.0 yr old M/F: NS | OE (n=38) | NACT (n=33) |
| ***Swaminathan et al. (2020)** ^54^ | RCT | IG (n=29) | Mean age: 56.03 yr old M/F: 18/11 | OG (n=29) | NS |
|  |  | CG (n=29) | Mean age: 56.82 yr old M/F: 20/9 | OG (n=29) | NS |
| ***Zylstra et al.** **(2020)** ^64^ | Cohort | IG (n=13) | Median age: 63.0 yr old  M/F: 12/1 | OG (n=12) | NACT (n=13) |
|  |  | CG (n=14) | Median age: 63.6 yr old  M/F: 13/1 | OG (n=13) | NACT (n=14) |
| **PERI- OR POSTOPERATIVE REHABILITATION** | | | | | |
| Fagevik Olsen et al. (2002) ^71^ | RCT | IG1 (n=36) | Mean age: 64.1 yr old M/F: 31/5 | OE (n=36) | NACRT (n=18) |
|  |  | IG2 (n=34) | Mean age: 62.0 yr old M/F: 29/5 | OE (n=34) | NACRT (n=19) |
| Nakamura et al. (2007)^69^ | Cohort Study | Total n=184 | Mean age: 64.0 yr old  M/F: 154/30 | OE or MIE (n=184) | Neo-adjuvant treatment total n=16 |
| Vats et al. (2009)^80^ | Cohort Study | IG 1 (n= 10 ) | Mean age: 53.4 yr old  M/F: 8/2 | Esophagectomy approach NS | NS |
|  |  | IG 2 (n= 10) | Mean age: 53.4 yr old  M/F: 7/3 |  |  |
|  |  | CG (n=10 ) | Mean age: 53.4 yr old  M/F: 7/3 |  |  |
| ***Lunardi et al. (2011)**^78^ | Cohort Study | IG (n= 40 ) | Mean age: 53 yr old M/F: 24/16 | OE (90%) MIE (10%) | NS |
|  |  | CG (n= 30) | Mean age: 56 yr old M/F: 17/13 | OE (90.1%) MIE (9.9%) | NS |
| ***Lococo et al. (2012)**^79^ | Cohort Study | IG (n= 8 ) | Mean age: 70 yr old  M/F: 6/2 | OE (n=8) | CT (n=2) or RT (n=4) NACT or NACRT (n=1) |
|  |  | CG (n= 50 ) | Mean age: 67.46yr old M/F: 34/16 | OE (n=50) | CT (n=14) or RT (n=22) NACT or NACRT (n=5) |
| ***Akiyama et al. (2017)**^72^ | Cohort Study | IG (n=31) | Mean age: 64.2 yr old M/F: 25/6 | OE (n=5) MIE (n=26) | NACT (n=14) NACRT (n=2) |
|  |  | CG (n= 21) | Mean age: 64.9 yr old M/F: 16/5 | OE (n=3) MIE (n=18) | NACT (n=11) NACRT (n=0) |
| ***Fagevik Olsen et al. (2017)**^66^ | RCT | IG (n=20) | Mean age: 62.7 yr old M/F: 16/4 | OE (n=20) | NS |
|  |  | CG (n=23) | Mean age: 62.3 yr old M/F: 19/4 | OE (n=23) | NS |
| ***Chen et al. (2017)**^67^ | RCT | IG (n=39) | Mean age: NS M/F: NS | OG or MIG (n=39) | NS |
|  |  | CG (n=41) | Mean age: NS M/F: NS | OG or MIG (n=41) | NS |
| ***O'Neill et al. (2018)**^69^ | RCT | IG (n=21) | Mean age: 67.19 yr old M/F: 17/4 | OE (n=19) OG (n=2) | NACT/NACRT/Periop CT (n=11) Adjuvant CRT or CT (n=4) |
|  |  | CG (n=22) | Mean age: 64.14 yr old M/F: 18/4 | OE (n=16) OG (n=6) | NACT/NACRT/Periop CT (n=16) Adjuvant CRT or CT (n=5) |
| Chang et al. (2019)^68^ | RCT | IG (n=44) | Mean age: 56 yr old M/F: 43/1 | OE (n=44) | NACT/NACRT (n=26) |
|  |  | CG (n=44) | Mean age: 56 yr old M/F: 37/7 | OE (n=44) | NACT/NACRT (n=24) |
| ***Jianjun et al. (2019)**^73^ | Cohort Study | IG (n=60) | Mean age: 61.1 yr old  M/F: 36/24 | OG (n=60) | Nil |
|  |  | CG (n=60) | Mean age: 63.1 yr old M/F: 38/22 | OG (n=60) | Nil |
| van Egmond et al. (2020)^75^ | Cohort Study | IG (n=15) | Mean age: 62.8 yr old M/F: 11/4 | OE (n=1) MIE (n=14) | NACRT (n=15) |
|  |  | CG (n=30) | Mean age: 60.3 yr old M/F: 22/8 | OE (n=1) MIE (n=29) | NACRT (n=30) |
| ***Wang et al. (2020)**^76^ | Cohort Study | IG (n=156) | Mean age: NS  M/F: 110/46 | OE (n=89)  MIE (n=67) | NS |
|  |  | CG (n=387) | Mean age: NS  M/F: 255/132 | OE (n=112)  MIE (n=275) | NS |
| Simonsen et al. (2020)^77^ | NRS | IG (n= 20) | Mean age: 63.7 yr old M/F: 17/3 | OE (n=16)  MIE (n=7) | NACRT (n=2)  CT (n=18) |
|  |  | CG (n=29) | Mean age: 66.0 yr old M/F: 27/2 | OE (n=17)  MIE (n=2) | NACRT (n=4)  CT (n=23) |
| ***Jiao et al. (2020)**^65^ | RCT | IG (n=43) | Mean age: 48.1 yr old M/F: 25/18 | MIE (n=43) | NS |
|  |  | CG (n=43) | Mean age: 46.7 yr old M/F: 27/16 | MIE (n=43) | NS |
| ***Van Vulpen et al. (2021)** | RCT | IG (n=54) | Mean age: 64.3 yr old  M/F: 52/9 | OE (n=5)  MIE (n=56) | (NA)CT (n=1)  (NA)CRT (n=51) |
|  |  | CG (n=56) | Mean age: 63.1 yr old  M/F: 52/7 | OE (n=6)  MIE (n=53) | (NA)CT (n=6)  (NA)CRT (n=47) |

**Abbreviations:** CG: control group; CT: adjuvant chemotherapy; HE: hybrid approach esophagectomy; IG: intervention group; MIG: minimally invasive gastrectomy; MIE: minimally invasive esophagectomy; NACRT: neo-adjuvant chemoradiotherapy; NACT: neo-adjuvant chemotherapy; NART: neo-adjuvant radiotherapy; NRS: non randomized controlled study; NS: not specified; OG: open gastrectomy; RCT: randomized controlled trial; OE: open esophagectomy

***** indicates studies included in the meta-analysis

Supplemental table S4: Details of interventions of all studies

| **Authors (year)** | **Study type** | **Group (n)** | **Intervention timing and duration** | **Intensity** | **Intervention components** |
| --- | --- | --- | --- | --- | --- |
| **PREHABILITATION** | | | | | |
| ***Dettling et al. (2012)**^63^ | NRS | IG (n=44) | - Timing: preoperatively respiratory training and postop physical therapy - Setting: 6 times per week unsupervised at home, 1 time per week in-hospital supervised by physiotherapist - Duration: min 2 weeks preop until day before surgery, followed by 10 days postop | - Resistance started at 30% of mouth pressure and increased by 10% every 3 mins based on Borg score (<5) - Trained daily, 7 times per week for at least 2 weeks  - 20 min per session | - Preop IMT: inspiratory threshold loading device - Usual care: postop physical therapy (deep breathing, coughing techniques and mobilization) |
|  |  | CG (n=39) | / | / | Usual care: postop physical therapy (deep breathing, coughing techniques and mobilization) |
| ***Inoue et al. (2013)**^9^ | Cohort study | IG (n=63) | - Timing: preoperatively - Setting: rehabilitation centre, supervised by physiotherapist and home-based training - Duration: min 7 days | - IMT with IS: 10 inspirations per set, 3 sets, 3 times a day - Biking: 15 mins - Program carried out for 40-60 min per day, during weekdays | Prehab: - IMT with IS - Respiratory muscles and thoracic cage stretching - Deep diaphragmatic breathing - Efficient coughing and huffing with vigorous contraction of abdominal muscles  - Muscle strength exercises for upper and lower limbs, and abdominal muscles - Biking on ergometer Outpatient:  - every day self‐training at home with IS training - Respiratory muscles and thoracic cage stretching, deep diaphragmatic breathing - efficient coughing and huffing Postop rehabilitation, from day 1: - Respiratory rehabilitation: positioning, respiratory muscles and thoracic cage stretching, deep diaphragmatic breathing, coughing and huffing and early mobilization until discharge |
|  |  | CG (n=37) | / | / | - Preop rehabilitation insufficiently (or not) received  - Postop rehabilitation from day 1: Respiratory rehabilitation: positioning, respiratory muscles and thoracic cage stretching, deep diaphragmatic breathing, coughing and huffing and early mobilization until discharge |
| ***Cho et al. (2014)**^60^ | Cohort study | IG (n=18) | - Timing: preoperatively - Setting: NS - Duration: 4 weeks | - Aerobic exercise: 3 to 7 days per week, intensity set according to maximal HR reserve or Borg score with expected energy expenditure of 30 kcal/kg/week - Resistance training: 1 to 2 times per week - Stretching before and after aerobic training | - Aerobic exercise: treadmill or bicycle ergometer, swimming, dancing, or jogging - Resistance training - Stretching before and after aerobic exercise |
|  |  | CG (n=54) | / | / | Usual care, no preop intervention |
| Van Adrichem et al. (2014)^56^ | RCT | IG1 (n=20) | - Timing: preoperatively until day before surgery - Setting: supervised, in-hospital - Duration: min 3 weeks | - 3 times per week - Resting time progressively reduced from 60 to 45, 30, 15 and 5 sec - Initial intensity 60% of MIP with increase to 80% in first week. Further sessions intensity 80% of MIP and increase with 5% if Borg score <5 | - IMT-HI: 6 cycles of 6 inspiratory manoeuvres on inspiratory threshold-loading device - Postop physical therapy: breathing exercises, coughing techniques, early mobilization |
|  |  | IG2 (n=19) | - Timing: preoperatively until day before surgery - Setting: supervised in-hospital and unsupervised home-based - Duration: min 3 weeks | - 7 times per week  - Initial intensity 30% of MIP with increase with 5% if Borg score <5 | - IMT-E: 20 min of breathing per sessions through inspiratory threshold-loading device - postop physical therapy: breathing exercises, coughing techniques, early mobilization |
| ***Yamana et al. (2015)**^49^ | RCT | IG (n=30) | - Timing: preoperatively - Setting: supervised, in-hospital - Duration: min 7 days | - 60 minutes daily, 5 times per week | - Respiratory muscle and thoracic cage stretching - Deep inspiration training and deep diaphragmatic breathing - Coughing and huffing - Muscle strength exercises LL and abdominal muscles - Biking on ergometer for 20 min - Postop rehabilitation until discharge: early mobilization, positioning, respiratory muscles and thoracic cage stretching, deep diaphragmatic breathing, coughing and huffing |
|  |  | CG (n=30) | / | / | - Usual care, no preop intervention - Postop rehabilitation until discharge: early mobilization, positioning, respiratory muscles and thoracic cage stretching, deep diaphragmatic breathing, coughing and huffing |
| Xu et al. (2015)^50^ | RCT | IG (n=28) | - Timing: preoperatively during NACRT  - Setting: supervised, in-hospital  - Duration: 4 to 5 weeks | - 3 times per week - Target HR using max HR formula ([220-age] x desired intensity of 60%) | - 5 min. warm-up  - 20 min. hallway ambulation |
|  |  | CG (n=28) | / | / | Usual care, no intervention |
| ***Weblin et al. (2017)**^59^ | NRS | IG (n= 13) | - Timing: preoperatively - Setting: supervised by physiotherapist and 1 unsupervised session at home - Duration: 4 weeks | Preop: - Twice weekly: 20 min 10 exercise stations, 1 min per station x2  - Intensity: 50-70% of HR reserve  - Borg breathlessness score 3 to 4 Postop:  - Twice per day for first 5 days and once per day from day 6 until discharge | - Preop exercises: within 1 to 2 weeks of completion of NACT and 1 week preop, circuit based with warm up and cool down, 10 exercises components (respiratory rehabilitation and mobilization) - ERAS and enhanced postop physiotherapy: pulmonary physiotherapy, mobilization and home exercise |
|  |  | CG (n=10) | - Timing: postoperatively  - Setting: in-hospital, reviewed once by a physiotherapist - Duration: up until postop day 10 | - Twice per day for first 5 days and once per day from day 6 until discharge | - ERAS and enhanced postop physiotherapy: pulmonary physiotherapy, mobilization and home exercise |
| ***Valkenet et al. (2017)**^55^ | RCT | IG (n=120) | - Timing: preoperatively, during neoadjuvant therapy - Setting: home-based, 1 individual session with physiotherapist and instruction video provided - Duration: min 2 weeks, until day of surgery | - 30 breathings 2 times per week for 7 days per week - Initial inspiratory load at 60% of baseline MIP and increased by 5% once Borg score <7 | - IMT with flow‐resistive inspiratory loading device - Postop physiotherapy from postop day 1: airway clearance technique and early mobilization - Adherence monitored by physiotherapist |
|  |  | CG (n=121) | / | / | - Usual care, no preop intervention - Postop physiotherapy from day 1: airway clearance technique and early mobilization |
| ***Christensen et al. (2018)**^57^ | NRS | IG (n= 21) | - Timing: preoperatively, during neoadjuvant treatment - Setting: in research centre, supervised - Duration: neoadjuvant treatment for approximately 9 weeks, surgery 4 to 6 weeks after completion of neoadjuvant therapy | - Aerobic and resistance training: high intensity, 2 times per week, 75 min session - Warm up: 10 min - High intensity interval training: 21 to 28 min - 3 min of low intensity recovery | - Aerobic and resistance (chest, leg press, lateral pull and knee extension, with 1 warm-up set followed by 3 sets of 8-12 repetitions) - Warm-up on stationary bicycle followed by high-intensity interval training 4x4 min with 3 min of low-intensity activity recovery |
|  |  | CG (n= 29 ) | / | / | Usual care: information about smoking cessation, diet, alcohol and physical activity guidelines |
| Herrstedt et al. (2018)^61^ | NRS | IG (n= 18 ) | - Timing: preoperatively - Setting: supervised - Duration: 12 weeks | - Aerobic exercise: twice weekly, 30-45 mins | - Exercise training: aerobic interval cycling on stationary biked - Resistance training with 4 exercises for major muscle groups (chest press, leg press, lateral pull and knee extension) |
|  |  | CG (n=25 ) | / | / | Usual care (nurse-led follow-up and information on lifestyle-related factors) or community based exercise programs |
| ***Minnella et al. (2018)**^51^ | RCT | IG (n=26) | - Timing: preoperatively - Setting: home-based, 1 individual session with physiotherapist - Duration: NS (77% during NACT) | - Aerobic exercises 3 times per week: intensity self-selected to reach 12 to 13 on Borg score. - Strengthening activity 1 time per week: 3 sets of 8 to 12 repetitions for 8 muscle groups using elastic band as resistance with level selected by kinesiologist to reach moderate-intensity effort. | - Aerobic exercises 30 min (incl. 5min warm-up and 5min cooldown) of moderate continuous training: brisk walk, jogging or cycling.  - Strengthening activity: 30min (incl. 5min flexibility and 5mins stretching).  - Nutrition: dietary advice and whey protein supplement for daily protein intake of 1.2 to 1.5g/kg of ideal body weight or 20% of total energy requirements.  - Adherence monitored by physiotherapist and nutritionist |
|  |  | CG (n=25) | / | / | Usual care: standardized ERAS protocol with early oral nutrition, respiratory physiotherapy and early mobilization |
| ***Guinan et al. (2018)**^53^ | RCT | IG (n=28) | Sub-cohort of PREPARE trial as described by Valkenet et al. (2017) | | |
|  |  | CG (n=32) | / | / | - Usual care, no preop intervention - Postop physiotherapy from day 1: airway clearance technique and early mobilization |
| ***Lam et al. (2018)**^52^ | RCT, Thesis | IG (n=5) | - Timing: preoperatively, during and after neoadjuvant therapy - Setting: home-based and in-hospital - Duration: 14 to 16 weeks | - IMT: 20min for 7 days a week with resistance at 30% of baseline MIP and increase by 5% if New Category Borg score < 5 - Advice: 30min, moderate intensity for 5 days per week or 20min vigorous intensity for 3 days per week - In-hospital: max 8 sessions with 2 sessions per week for 4 weeks of aerobic (4min warm-up light intensity cycling, Borg score 9-11, followed by 30min moderate intensity cycling, Borg score 12-13) and muscle strengthening exercise (2min per set) | - Home based: IMT during and after NACT, using inspiratory threshold loading device and home exercise advice - Usual care: home exercise advice - In-hospital: aerobic (cycling) and muscle strengthening exercise - Adherence monitored with exercise diary |
|  |  | CG (n=6) | / | - Advice: 30min, moderate intensity for 5 days per week or 20 min vigorous intensity for 3 days per week | Usual care: home exercise advice |
| ***Akiyama et al. (2020)**^62^ | Cohort Study | IG (n=23) | - Timing: preoperatively - Setting: in-hospital, supervised by physiotherapist - Duration: 7 days | - Aerobic exercise: 20 to 30 mins, twice per day, at 60-70% max HR - Muscle strength training: 20 sets of squats, each performed 10 to 15 times, twice per day | - Aerobic exercise: cycling ergometer - Muscle strength training: squatting - Usual care: 1 month preop, home-based IMT, unsupervised; encouraged to walk for 20 mins and perform squats; postop day 1 in-hospital, supervised: early mobilization with ambulation, respiratory training and training for chewing and swallowing; after postop day 5: exercise training consisting of cycling ergometer, IMT with IS and walking |
|  |  | CG (n=25) | / | Usual care: - IMT: 4 to 5 sets of 10 deep inspirations per day - Squats: 20 sets, 10 times per day - Postop day 5 exercise training: twice per day, cycling ergometer for 20 to 30mins, 2 sessions each component per day | - Historical controls  - Usual care: 1 month preop, home-based IMT, unsupervised; encouraged to walk for 20 mins and perform squats; postop day 1 in-hospital, supervised: early mobilization with ambulation, respiratory training and training for chewing and swallowing; after postop day 5: exercise training consisting of cycling ergometer, IMT with IS and walking |
| ***Halliday et al. (2020)**^58^ | Cohort Study | IG (n=38) | Timing: preoperatively  Setting: home-based  Duration: NS, during neo-adjuvant treatment | - Exercise training: min 600 MET minutes per week, aim to increase to 1200 MET minutes per week  - Borg score target 13-15 | - Aerobic and strength exercise training  - Weekly telephone follow-up by physiotherapist  - Nutritional support: plan based on nutritional risk and dietary eating habits, weekly or fortnightly follow-up by dietician  - Psychological support  - ERAS incl. early mobilization |
|  |  | CG (n=38) | / | / | ERAS incl. early mobilization |
| ***Swaminathan et al. (2020)** ^54^ | RCT | IG (n=29) | - Timing: preoperatively - Setting: home-based - Duration: 7 days | IS: 15 times per sessions, every 4 hours | - Volume-orientated IS - ERAS protocol, incl. oral maltodextrin drink night before and day of surgery, and ambulation from postop day 1 |
|  |  | CG (n=29) | / | / | Usual care, no preop intervention |
| ***Zylstra et al**. **(2020)**^64^ | Cohort | IG (n=13) | - Timing: preoperatively  - Setting: clinic, supervised by physiotherapist  - Duration: NS, during neo-adjuvant treatment | - Aerobic exercises: 30 min  - Strength exercises: 12 exercises, 3 sets  - Core strength and stability training: 7 exercises, 3 sets  - Flexibility exercises | - Aerobic exercises: 4weeks progressive walking  - Strength exercises using band to increase resistance  - Flexibility exercises consisting of stretching, warm-up and cool-down performed slowly |
|  |  | CG (n=14) | / | / | No preop intervention |
| **PERI- OR POSTOPERATIVE REHABILITATION** | | | | | |
| Fagevik Olsen et al. (2002) ^71^ | RCT | IG1 (n=36) | - Timing: immediate postoperatively - Setting: in-hospital - Duration: NS, from postop day 1 | - 30 deep breaths with huffing and coughing between every 10th breath at 2h intervals - Resistance: -5 cmH20 during inspiration and +10 cmH_2_0 during expiration | - IR-PEP: deep breathing exercises with PEP mask with T-valve dividing inspiration and expiration |
|  |  | IG2 (n=34) | - Timing: immediate postoperatively - Setting: in-hospital - Duration: from postop day 1-3, then continued with IR-PEP intervention | - 30min every 2h - Resistance: 5-10 cmH20 | CPAP |
| Nakamura et al. (2007)^69^ | Cohort Study | Total n=184 | - Timing: perioperatively, aerobic exercise 2 weeks before surgery, and from day 1 postop - Setting: home-based and in-hospital with a physiotherapist - Duration: NS | NS | - Respiratory physiotherapy: breathing exercises with pursed lips, huffing, coughing, abdominal respiration, and expectoration  - Systemic exercises: riding a bicycle and walking, 2 weeks preoperatively  - Deep breathing with huffing and coughing and postural drainage to assist breathing and expectoration, from postop day 1 |
|  |  |  | / | / | Historical controls |
| Vats et al. (2009)^80^ | Cohort Study | IG 1 (n= 10) | - Timing: immediate postoperatively - Setting: in-hospital - Duration: until discharge | - IS: each hour for 8 waking hours, 15 repetitions of max inspiration each hour for 5 days | - IS device (2 balls) 1200 cubic cm, max inhalation, hold inspiration for 5sec, complete exhalation |
|  |  | IG 2 (n= 10) | - Timing: immediate postoperatively - Setting: in-hospital - Duration: until discharge | - Deep breathing exercises: 2 times in an interval of 6 hours, for 15 repetitions for 5 days | - Diaphragmatic, segmental, posterior basal, apical and pursed lip breathing exercises - Right middle lobe or lingual expansion - Mobilization of upper chest and shoulders - Wand and coughing exercises - Splinting - Active ROM exercises to shoulders and trunk for chest expansion, deep breathing and stimulation of cough reflex - Humidification and nebulisation |
|  |  | CG (n=10) | / | / | Usual care, no postop intervention |
| ***Lunardi et al. (2011)**^78^ | Cohort Study | IG (n= 40) | - Timing: postoperatively - Setting: in-hospital with physiotherapist  - Duration: until discharge | - Exercises associated with max sustained and fractional inspiration - <1 session daily - 20 min per session | - Lung re-expansion, airway clearance manoeuvres (assisted cough and expiratory airflow techniques) and early mobilization |
|  |  | CG (n= 30) | / | / | Historical controls |
| ***Lococo et al. (2012)**^79^ | Cohort Study | IG (n= 8) | - Timing: postoperatively - Setting: multidisciplinary (physiotherapist, physician, nurse, psychologist, dietician), supervised and unsupervised - Duration: 4 weeks | - 5 daily sessions each week for 4 weeks - 2-weekly educational session | - Supervised symptom-limited incremental exercise (cyclo-ergometer test) - Abdominal muscle activities, inspiratory resistive sessions, treadmill, upper and lower extremities training and full arm circling - Educational sessions 2x/week, dietary counselling, relaxation and stress management techniques, energy conservation principles and breathing retraining |
|  |  | CG (n=50) | / | / | - Historical controls  - Chest physiotherapy: coughing, deep breathing, abdominal breathing - General exercise therapy - Specific training session for inspiratory muscles to achieve early mobilization |
| ***Akiyama et al. (2017)**^72^ | Cohort Study | IG (n=31) | - Timing: perioperatively - Setting: In-hospital - Duration: NS | NS | - Education by physiotherapist 1month preop - Postop: IMT and early mobilization from day 1, walking from day 4, muscle strength and ergometer training after day 7 |
|  |  | CG (n= 21) | / | / | Historical controls |
| ***Fagevik Olsen et al. (2017)**^66^ | RCT | IG (n=20) | - Timing: postoperatively at discharge - Setting: first program supervised, remaining programs unsupervised home-based - Duration: 3 months | - 10 repetitions of each exercise daily - Gradual increase in intensity throughout rehabilitation | - Standardized physiotherapy postop day 1: breathing exercises with CPAP during ICU, followed by deep breathing exercises with PEP every 2h during daytime, and mobilization - Postop intervention: 3 exercise programs to restore lung function, ROM in thoracic spine and shoulder, and strength exercises for back extensors, shoulders, and legs |
|  |  | CG (n=23) | / | / | Usual care: given information to avoid specific interventions during first 3 months postop |
| ***Chen et al. (2017)**^67^ | RCT | IG (n=39) | - Timing: immediate postoperatively  - Setting: in-hospital - Duration: until discharge | NS | - Early mobilization  - Nutritional assistance: education, encourage oral intake and feeding assistance if needed  - Nurse-led |
|  |  | CG (n=41) | / | / | Usual care: mobilization encouraged, not enforced |
| ***O'Neill et al. (2018)**^69^ | RCT | IG (n=21) | - Timing: postoperatively, in long-term survivors - Setting: supervised and unsupervised (home-based) exercise  - Duration: 12 weeks | - Aerobic exercise: start at low intensity (30-45% HR reserve), progressed weekly to moderate intensity (45-60%) - Resistance: start at 2 sets of 12 repetition max, progressed to 6 of 17 repetition max | - Aerobic exercise: treadmill walking, stationary cycling and cross training in supervised sessions; walking or stationary cycling during home-based sessions - Resistance training: free weights and horizontal leg press in supervised sessions; use of TheraBands during home-based sessions - Adherence monitored using polar HR monitors and exercise diaries - Dietary counselling: personalised diet-related advice and individual goals |
|  |  | CG (n=22) | / | / | Usual care |
| Chang et al. (2019)^68^ | RCT | IG (n=44) | - Timing: postoperatively * Setting: home-based, instructions given before hospital discharge - Duration: 12 weeks | - 30min, 3 to 5 days per week or total of 150min per week - Moderate intensity, aim Borg score of 12 to 16, HR reserve percentage of 55-65% | - Walking exercise after meals, monitored by smart bracelet; adherence monitored by online platform data upload - Nursing education program (e-books: diet guidance, rehabilitation exercises, symptom management, psychological adjustments) - Regular postoperative rehabilitation exercises |
|  |  | CG (n=44) | / | NS | - Usual care: regular postop rehabilitation exercises on daily basis |
| ***Jianjun et al. (2019)**^73^ | Cohort Study | IG (n=60) | - Timing: perioperatively, 7 days preop  - Setting: NS - Duration: 7 days | - Daily endurance training - Walking min 3000 meters daily - Climbing at least 8 floors per time - Breathing: 2 to 3 times per day > 10 min at a time; 5 second balloon blow and hold | - Encourage preop physical exercise and endurance training: daily walking; climbing; breathing exercises (effective coughing, blow balloon) - Preop nutritional risk screening: oral enteral Nutrison of 0.5 barrels (1000 kcal) per day if risk for malnutrition - Improve awareness of disease and treatment compliance |
|  |  | CG (n=60) | - Timing: day 3 postoperatively - Setting: in-hospital - Duration: until discharge | - Walking min 30 minutes daily | - Postop rapid rehabilitation: encourage walking; dietary instruction |
| van Egmond et al. (2020)^75^ | Cohort Study | IG (n=15) | - Timing: postoperatively, from discharge - Setting: home-based, weekly follow-up by physiotherapist via telephone, e-mail or video-sessions -Duration: 12 weeks | - Functional exercises: intensity and frequency according to guidelines of the American College of Sports Medicine -Cardiorespiratory exercises: 2 or more sessions per week, moderate-to-vigorous intensity level (Borg score 6-20) - Muscle strength exercises: 2 to 3 days per week, moderate-to-hard intensity (60 to 70% of the 1 repetition maximum) using Holten curve that relates the percentage of the 1 repetition maximum to the estimated repetitions of that intensity | - Telerehabilitation intervention: eHealth platform Physitrack, to improve functional activity level by increasing muscle strength, coordination, ROM and stamina |
|  |  | CG (n=30) | / | / | Historical controls |
| ***Wang et al. (2020)**^76^ | Cohort Study | IG (n=156) | - Timing: perioperatively  - Setting: in-hospital, supervised by physiotherapist  - Duration: until discharge | - Expiratory flow rate training: 5 to 8 times per session, 2-3 sessions per day  - hand-assisted sputum excretion: 3 deep breaths | - Preop pulmonary rehabilitation education  - Preop rehabilitation for 3 to 7 days preop: respiratory muscle training (abdominal and lip constriction breathing); expiratory flow rate training; cough method training (deep breath, hold breath and coughing); board training (adjusting breathing rhythm during exercise, supervised by physiotherapist)  - Postop: early mobilization, respiratory muscle training (deep breathing); airway clearance techniques (active cycle of breathing techniques, mechanically assisted sputum discharge and buckle vibrations); hand-assisted sputum excretion aided by physiotherapist; administration of Ambroxol and Doxofylline |
|  |  | CG (n=387) | / | / | - Preop pulmonary rehabilitation education  - Usual care, without preop or postop rehabilitation |
| Simonsen et al. (2020)^77^ | NRS | IG (n= 20) | - Timing: from 6 weeks postoperatively - Setting: in-hospital, supervised - Duration: 12 weeks | - Aerobic exercise: 10mins warm-up at 60-70% max HR, followed by 4 min high intensity interval aiming to reach min 75% max HR for sessions 1 to 4, and 85 to 95% from session 5 - Resistance training: 3 sets, load set to 50 to 60% of 1-repetition max test during sessions 1 to 4, to 60 to 70% for sessions 5 to 12, and to 70 to 80% for sessions 13 to 24 | - Aerobic exercise: bicycle or treadmill - Resistance training: leg press, leg extension, seated row and chest press |
|  |  | CG (n=29) | / | / | Usual care: allowed to exercise on their own and to take part in other exercise programs |
| ***Jiao et al. (2020)**^65^ | RCT | IG (n=43) | - Timing: perioperatively (pre- and postop) - Setting: in-hospital and home-based - Duration: NS | - Deep breathing: 10 to 20 times per group, 2 groups per day - Abdominal and pursed-lips breathing: 10 to 15 min each time, 3 times per day - Atomization inhalation: 3 to 4 times per day, 15 to 20 min each time | - Preop respiratory function training: deep breathing exercises with balloons; abdominal and pursed-lips breathing training - Sputum elimination: lean forward, slow deep breaths, abdominal muscles contraction and continuous cough for 2 to 3 times while breath-holding, cough effectively - Atomization inhalation if necessary - Postop dietary guidance: NG feeding during first 3 days, gradually receiving high protein, high vitamin, high calorie and digestible food - Weekly telephone FU after discharge to monitor diet, exercise and respiratory quality |
|  |  | CG (n=43) | / | / | Usual care, no periop physiotherapy intervention |
| ***Van Vulpen et al. (2021)** ^70^ | RCT | IG (n=54) | - Timing: postoperatively  - Setting: in-hospital or in a general physiotherapist practice, supervised by physiotherapist, and home-based  - Duration: 12 weeks | - Aerobic exercise: 50 min with increasing intensity every 3 weeks (based upon HR reserve)  - Resistance training: 1 set of 20-25 repetitions at 20-RM weight during the first 8 weeks, followed by 2 sets of 15-20 repetitions at 15-RM weight  - Warm-up and cool-down | - Aerobic exercise: treadmill, bicycle or cross-trainer; in the last 3 weeks performing additional 10  sets of 30 sec vigorous to maximal exercise, alternated with 1 min active rest  - Resistance training: rowing, bench press, squat, shoulder press, biceps curl, lunges, calf-raises, triceps extension and abdominal crunch/hoover |
|  |  | CG (n=56) | / | / | / |

**Abbreviations:** CG: control group; CPAP: continuous positive airway pressure; HR: heart rate; IG: intervention group; IMT: inspiratory muscle training; IMT-E: inspiratory muscle training-endurance ;IMT-HI: inspiratory muscle training-high intensity; IR-PEP: inspiratory resistance-positive expiratory pressure; IS: incentive spirometer; LL: lower limbs; MET: metabolic equivalent for task; NRS: non randomized controlled study; NS: not specified; PEF: peak expiratory flow; PEP: positive expiratory pressure; PIF: peak inspiratory flow; RCT: randomized controlled trial; RM: repetition maximum; ROM: range of motion; UL: upper limbs

***** indicates studies included in the meta-analysis

Supplemental table S5: Statistical method used for each analysis

| **Prehabilitation** | | | | | | | |
| --- | --- | --- | --- | --- | --- | --- | --- |
| **Outcome** | **Sample size** | **Sample size IG (events)** | **Sample size CG (events)** | **Statistical heterogeneity** | **Statistical model** | **Outcome measure** | **p-value** |
| Functional exercise capacity (6MWT) | 159 | 77 | 82 | High (I^2^=94.0%;p<0.00001) | Random-effects | Pooled mean difference 26.70, 95% CI -73.10 to 126.49 | p= 0.60 |
| Incidence of pneumonia, including combined incidence of pneumonia and other PPC | 828 | 403 (103) | 425 (132) | Moderate ((I^2^ = 50%, p=0.03) | Fixed-effects | Pooled OR = 0.70; 95% CI 0.51 to 0.95 | p=0.02 |
| Incidence of pneumonia, excluding the combined incidence of pneumonia and other PPC | 757 | 370 (91) | 387 (117) | Moderate (I^2^ = 59%, p=0.01) | Fixed-effects | Pooled OR = 0.68; 95% CI 0.49 to 0.95 | p=0,02 |
| Incidence of other PPC, including the combined incidence of pneumonia and other PPC | 588 | 274 (78) | 314 (104) | Moderate (I^2^ = 57%, p=0.03) | Fixed-effects | Pooled OR = 0.73; 95% CI 0.51 to 1.05 | p=0,09 |
| Incidence of other PPC, excluding the combined incidence of pneumonia and other PPC | 517 | 241 (66) | 276 (89) | Moderate (I^2^ = 71%, p=0.008) | Fixed-effects | Pooled OR = 0.71; 95% CI 0.48 to 1.05 | p=0.09 |
| Incidence of postoperative morbidity | 468 | 241 (52) | 227 (91) | No statistical heterogeneity (I² = 0%, p=0.48) | Fixed-effects | Risk difference = -0.16; 95% CI -0.24 to -0.09 | p<0.0001 |
| In-hospital mortality | 570 | 284 (6) | 286 (6) | Low (I² = 41%, p=0.19) | Fixed-effects | Pooled OR = 0.97, 95% CI 0.31 to 3.03 | p=0.95 |
| LOS | 889 | 436 | 453 | High (I² = 80%, p<0.00001) | Random-effects | Mean difference = -0.44, 95% CI -1.69 to 0.82 | p=0.50 |
| **Rehabilitation** | | | | | | | |
| **Outcome** | **Sample size** | **Sample size IG (events)** | **Sample size CG (events)** | **Heterogeneity** | **Statistical model** | **Outcome measure** | **p-value** |
| Incidence of pneumonia, including combined incidence of pneumonia and other PPC | 427 | 196 (16) | 231 (47) | No statistical heterogeneity (I^2^=0%, p=0.93) | Fixed-effects | Pooled OR = 0.42; 95% CI 0.20 to 0.86 | p=0.02 |
| Incidence of pneumonia, excluding the combined incidence of pneumonia and other PPC | 369 | 188 (14) | 181 (34) | No statistical heterogeneity (I^2^ = 0%, p=0.99) | Fixed-effects | Pooled OR = 0.35; 95% CI 0.16 to 0.78 | p=0.01 |
| Incidence of other PPC, including the combined incidence of pneumonia and other PPC | 307 | 136 (25) | 171 (33) | No statistical heterogeneity (I^2^ = 0%, p=0.42) | Fixed-effects | Pooled OR = 1.18; 95% CI 0.60 to 2.32 | p=0.63 |
| Incidence of other PPC, excluding the combined incidence of pneumonia and other PPC | 249 | 128 (23) | 121 (20) | No statistical heterogeneity (I^2^ = 20%, p=0.29) | Fixed-effects | Pooled OR = 1.23; 95% CI 0.59 to 2.58 | p=0.58 |
| LOS | 365 | 190 | 175 | No statistical heterogeneity (I² = 0%, p=0.75) | Fixed-effects | Mean difference = -1.74, 95% CI -2.89 to -0.59 | p=0.003 |
| HRQoL Summary score | 196 | 95 | 101 | Low (I² = 29%, p=0.24) | Fixed-effects | Mean difference 0.35, 95% CI -2.60 to 3.30 | p=0.82 |
| HRQoL Global Health | 196 | 95 | 101 | Low (I² = 14%, p=0.31) | Fixed-effects | Mean difference 0.45, 95% CI -4.61 to 5.50 | p=0.86 |
| HRQoL dyspnea | 153 | 74 | 79 | No statistical heterogeneity (I² = 0%, p=0.93) | Fixed=effects | Mean difference -8.53, 95% CI -15.14 to -1.91 | p=0.01 |
| HRQoL physical functioning | 196 | 95 | 101 | No statistical heterogeneity (I² = 0%, p=0.72) | Fixed-effects | Mean difference 5.14, 95% CI 1.23 to 9.05 | p=0.01 |
| HRQoL fatigue | 196 | 95 | 101 | No statistical heterogeneity (I² = 0%, p=0.76) | Fixed-effects | Mean difference -1.90, 95% CI -7.72 to 3.93 | p=0.52 |
| HRQoL pain | 153 | 74 | 79 | No statistical heterogeneity (I² = 0%, p=0.78) | Fixed-effects | Mean difference -3.81, 95% CI -10.00 to 2.39 | p=0.23 |

**Abbreviations:** 6MWT: 6-minute walking test; CI: confidence interval; CG: control group; HRQoL: Health-Related Quality of Life; IG: intervention group; LOS: length of hospital stay; OR: odds ratio; PPC: postoperative pulmonary complications

Supplemental table S6: Included and excluded studies for each analysis

| **Prehabilitation** | | | |
| --- | --- | --- | --- |
| **Outcome** | **Included studies** | **Excluded studies** | **Reason for exclusion** |
| Functional exercise capacity | Minnella et al. (2018)^51^, Guinan et al. (2018)^53^, Akiyama et al. (2020)^62^ measured 6MWT postoperatively | Xu et al. (2015)^50^, Lam et al. (2018)^52^, Christensen et al (2018)^57^, Weblin et al. (2017)^59^, Zylstra et al. (2020)^64^ | - Performed incremental exercise test^59^ or CPET^52, 57, 64^  - Measured 6MWT preoperatively^50^ |
| Incidence of pneumonia | Inoue et al. (2013)^9^, Yamana et al. (2015)^49^, Lam et al. (2018)^52^, Guinan et al. (2018)^53^, Valkenet et al. (2017)^55^, Christensen et al (2018)^57^, Halliday et al. (2020)^58^, Cho et al. (2014)^60^, Akiyama et al. (2020)^62^, Dettling et al. (2012)^63^, Zylstra et al. (2020)^64^ | None | / |
| Incidence of pneumonia only | Inoue et al. (2013)^9^, Yamana et al. (2015)^49^, Valkenet et al. (2017)^55^, Christensen et al (2018)^57^, Halliday et al. (2020)^58^, Cho et al. (2014)^60^, Akiyama et al. (2020)^62^, Dettling et al. (2012)^63^, Zylstra et al. (2020)^64^ | Lam et al. (2018)^52^, Guinan et al. (2018)^53^ | Reporting the combined incidence of pneumonia and other PCC^52, 53^ |
| Incidence of other PPC | Lam et al. (2018)^52^, Guinan et al. (2018)^53^, Valkenet et al. (2017)^55^, Halliday et al. (2020)^58^, Cho et al. (2014)^60^, Akiyama et al. (2020)^62^, Dettling et al. (2012)^63^ | None | / |
| Incidence of other PPC only | Valkenet et al. (2017)^55^, Halliday et al. (2020)^58^, Cho et al. (2014)^60^, Akiyama et al. (2020)^62^, Dettling et al. (2012)^63^ | Lam et al. (2018)^52^, Guinan et al. (2018)^53^ | Reporting the combined incidence of pneumonia and other PPC^52, 53^ |
| Postoperative morbidity | Inoue et al. (2013)^9^, Yamana et al. (2015)^49^, Minnella et al. (2018)^51^, Swaminathan (2020)^54^, Christensen et al (2018)^57^, Halliday et al. (2020)^58^, Akiyama et al. (2020)^62^, Zylstra et al. (2020)^64^ | None | / |
| Mortality | Yamana et al. (2015)^49^, Minnella et al. (2018)^51^, Guinan et al. (2018)^53^, Valkenet et al. (2017)^55^, Akiyama et al. (2020)^62^, Dettling et al. (2012)^63^, Zylstra et al. (2020)^64^ | None | / |
| LOS | Inoue et al. (2013)^9^, Minnella et al. (2018)^51^, Guinan et al. (2018)^53^, Swaminathan (2020)^54^, Valkenet et al. (2017)^55^, Akiyama et al. (2020)^62^, Christensen et al (2018)^57^, Halliday et al. (2020)^58^, Weblin et al. (2017)^59^, Cho et al. (2014)^60^, Dettling et al. (2012)^63^, Zylstra et al. (2020)^64^ | None | / |
| **Peri- or postoperative rehabilitation** | | | |
| **Outcome** | **Included studies** | **Excluded studies** | **Reason for exclusion** |
| Functional exercise capacity | None due to small number of studies (n=2) measuring 6MWT | Chang et al. (2020)^68^, O’Neill et al. (2018)^69^, Van Egmond et al. (2020)^75^,  Simonsen et al. (2020)^77^, Lococo et al. (2012)^79^ | - Performed incremental exercise test^77^, CPET^68,69^ or 2MWT^75^  - Only two studies measured 6MWT^68,79^ |
| Incidence of pneumonia | Jiao et al. (2020)^65^, Akiyama et al. (2017)^72^, Jianjun et al. (2019)^73^, Wang et al. (2020)^76^, Lunardi et al. (2011)^78^, Lococo et al. (2012)^79^ | None | / |
| Incidence of pneumonia only | Jiao et al. (2020)^65^, Akiyama et al. (2017)^72^, Jianjun et al. (2019)^73^, Wang et al. (2020)^76^, Lunardi et al. (2011)^78^ | Lococo et al. (2012)^79^ | Reporting the combined incidence of pneumonia and other PCC^79^ |
| Incidence of other PPC | Jiao et al. (2020)^65^, Akiyama et al. (2017)^72^, Wang et al. (2020)^76^, Lunardi et al. (2011)^78^, Lococo et al. (2012)^79^ | None | / |
| Incidence of other PPC only | Jiao et al. (2020)^65^, Akiyama et al. (2017)^72^, Wang et al. (2020)^76^, Lunardi et al. (2011)^78^ | Lococo et al. (2012)^79^ | Reporting the combined incidence of pneumonia and other PPC^79^ |
| LOS | Fagevik Olsen et al.(2017)^66^, Chen et al. (2017)^67^, Akiyama et al. (2017)^72^, Jianjun et al. (2019)^73^, Lunardi et al. (2011)^78^ | None | / |
| HRQoL | Lam et al. (2018)^52^, Swaminathan (2020)^54^, Akiyama et al. (2020)^62^, Zylstra et al. (2020)^64^, Fagevik Olsen et al.(2017)^66^, Chang et al. (2020)^68^, O’Neill et al. (2018)^69^, Van Vulpen et al. (2021)^70^, Nakamura et al. (2008)^75^ | Lam et al. (2018)^52^, Swaminathan (2020)^54^, Akiyama et al. (2020)^62^, Zylstra et al. (2020)^64^, Chang et al. (2020)^68^, Nakamura et al. (2008)^75^ | - Reporting summary score only^52^  - Reporting changes in HRQoL^75^, value at specific time point not provided  - Reporting changes in HRQoL in comparison with control group as reference^68^  - Only two studies reported HRQoL after prehabilitation^52, 64^  - Reporting HRQoL at different time points^54, 62^ |

**Abbreviations:** 2MWT: 2-minute walking test; 6MWT: 6-minute walking test; CPET: cardiopulmonary exercise testing; HRQoL: Health-Related Quality of Life; LOS:

length of hospital stay; PPC: postoperative pulmonary complications

Supplemental table S7: Newcastle-Ottawa Quality Assessment Scale for Cohort Studies

| **Author (year)** | **Selection** | **Comparability** | **Outcome** |
| --- | --- | --- | --- |
| Lunardi et al. (2011)^63^ | **🟑🟑🟑** | **🟑** | **🟑🟑** |
| Lococo et al. (2012)^79^ | **🟑🟑** | **🟑** | **🟑🟑** |
| Dettling et al. (2012)^63^ | **🟑🟑🟑🟑** | **🟑🟑** | **🟑🟑🟑** |
| Inoue et al. (2013)^9^ | **🟑🟑🟑🟑** | **🟑🟑** | **🟑🟑** |
| Cho et al. (2014)^60^ | **🟑🟑🟑🟑** | **🟑** | **🟑🟑** |
| Weblin et al. (2017)^59^ | **🟑🟑🟑🟑** | - | **🟑🟑** |
| Akiyama et al. (2017)^72^ | **🟑🟑** | **🟑🟑** | **🟑🟑** |
| Christensen et al. (2018)^57^ | **🟑🟑🟑🟑** | **🟑🟑** | **🟑🟑🟑** |
| Jianjun et al. (2019)^73^ | **🟑🟑🟑** | **🟑** | **🟑🟑** |
| Halliday et al. (2020)^58^ | **🟑🟑🟑** | **🟑🟑** | **🟑🟑** |
| Akiyama et al. (2020)^62^ | **🟑🟑🟑** | **🟑🟑** | **🟑🟑** |
| Wang et al. (2020)^76^ | **🟑🟑🟑** | **🟑🟑** | **🟑🟑** |
| Zylstra et al. (2020)^64^ | **🟑🟑🟑** | **🟑🟑** | **🟑🟑** |
